# Well-defined diatomic catalysis for photosynthesis of C_2_H_4_ from CO_2_

**DOI:** 10.1038/s41467-024-46745-3

**Published:** 2024-03-18

**Authors:** Zhongkai Xie, Shengjie Xu, Longhua Li, Shanhe Gong, Xiaojie Wu, Dongbo Xu, Baodong Mao, Ting Zhou, Min Chen, Xiao Wang, Weidong Shi, Shuyan Song

**Affiliations:** 1 School of Chemistry and Chemical Engineering, Jiangsu University, Zhenjiang, 212013 China; 2 State Key Laboratory of Rare Earth Resource Utilization, Changchun Institute of Applied Chemistry, Chinese Academy of Sciences, Changchun, 130022 China

**Keywords:** Energy, Structural properties, Natural gas, Photocatalysis

## Abstract

Owing to the specific electronic-redistribution and spatial proximity, diatomic catalysts (DACs) have been identified as principal interest for efficient photoconversion of CO_2_ into C_2_H_4_. However, the predominant bottom-up strategy for DACs synthesis has critically constrained the development of highly ordered DACs due to the random distribution of heteronuclear atoms, which hinders the optimization of catalytic performance and the exploration of actual reaction mechanism. Here, an up-bottom ion-cutting architecture is proposed to fabricate the well-defined DACs, and the superior spatial proximity of CuAu diatomics (DAs) decorated TiO_2_ (CuAu-DAs-TiO_2_) is successfully constructed due to the compact heteroatomic spacing (2-3 Å). Owing to the profoundly low C-C coupling energy barrier of CuAu-DAs-TiO_2_, a considerable C_2_H_4_ production with superior sustainability is achieved. Our discovery inspires a novel up-bottom strategy for the fabrication of well-defined DACs to motivate optimization of catalytic performance and distinct deduction of heteroatom synergistically catalytic mechanism.

## Abstract (web summary)

Principal interest lies in diatomic catalysts (DACs) for efficient CO2 to C2H4 photoconversion yet optimizing catalytic performance and exploring reaction mechanisms are impeded by heteronuclear atom random distribution. Here, a novel up-bottom ion-cutting architecture is proposed for fabricating well-defined DACs.

## Introduction

Photocatalytic carbon dioxide (CO_2_) reduction, a mimicking natural photosynthesis, is identified as an ideal technology to reduce the CO_2_ level by the conversion of CO_2_ into fuels or industrial feedstocks with the utilization of solar energy^1–3^. Among all the photoreduction CO_2_ products, ethylene (C_2_H_4_) is considered as high-value species due to their high energy densities and commercial prices in chemical industry^4–13^. Bimetallic solid-photocatalysts, consisted of ordered stagger of heteronuclear metal atoms, can strikingly reduce C-C coupling energy barrier via weakening dipole-dipole repulsion of the neighboring adsorbed C-based intermediates, which has been regarded as the dominator in photosynthesis of C_2_H_4_^14–21^. Nevertheless, the compact atom stacking during the synthesis of bimetallic solid catalysts inevitably contributes to the decrease of active sites^22–27^, critically restricting the efficient C_2_H_4_ generation. Therefore, its deep desirability to accurately optimize bimetallic solid catalysts at the atomic scale to break the bottleneck of inherent scaling-catalytic relationship in photosynthesis of C_2_H_4_.

Single-atom catalysts (SACs), a well-defined mononuclear metal sites, which have attracted huge attention for their potential to overcome the disadvantages of previously developed solid catalysts^27^. Conspicuously, heteronuclear DACs can not only maintain the maximized atom utilization but also modulate the reaction kinetics and even the reaction pathways by involving two metal atoms with cooperative modification of their steric and electronic properties^26–31^. So far, all reported heteronuclear DACs fabrication have concentrated on bottom-up synthetic strategies, such as organometallic compounds pyrolysis^26,32–35^, metal complexation^36–38^, metal coprecipitation^39–43^, in situ photoreduction^44,45^, and physical desorption^46,47^. Because of the repellency between the bare metal atoms under such prevailing bottom-up synthesis methods, disordered heteronuclear sites distribution have been an insurmountable barrier, which directly restrained the optimization of catalytic performance and distinct deduction of catalytic mechanisms^48^. Consequently, it is of high priority to exploit novel strategy for well-defined heteronuclear DACs fabrication to establish a high-efficiency photosynthesis of C_2_H_4_ system.

Here, an up-bottom ion-cutting engineering for the synthesis of atomic-level catalysts was initially proposed. Depending on the vectored etching of Cu in a CuAu alloy (isolated Au atoms in the Cu lattice), the CuAu-DAs with subnanometer heteroatomic spacing supported by commercial TiO_2_ (CuAu-DAs-TiO_2_) were successfully fabricated. The C_2_H_4_ production of CuAu-DAs-TiO_2_ proceeded at a remarkable rate of 568.8 μmol·g^−1^·h^−1^ without any sacrificial agent, which is superior to recent reported works in photoconversion CO_2_ and H_2_O into C_2_H_4_ (Supplementary Table 1), and no apparent catalyst deactivation was observed during the 120-h photocatalytic stability test. In such CuAu-DAs structures, Cu single atoms (Cu-SAs) are mainly responsible for the high-efficiency *CO generation rather than C-C coupling centers^16,17,49–52^, while Au single atoms (Au-SAs) serve as *CO coupling centers to rapidly consume the *CO arising from Cu-SAs according to photocatalytic CO_2_ reduction, which synergistically promotes the high efficiency and sustainability of photoconversion of CO_2_ into C_2_H_4_^53^.

## Results

### Catalyst synthesis and structural characterization

**Fig. 1:**
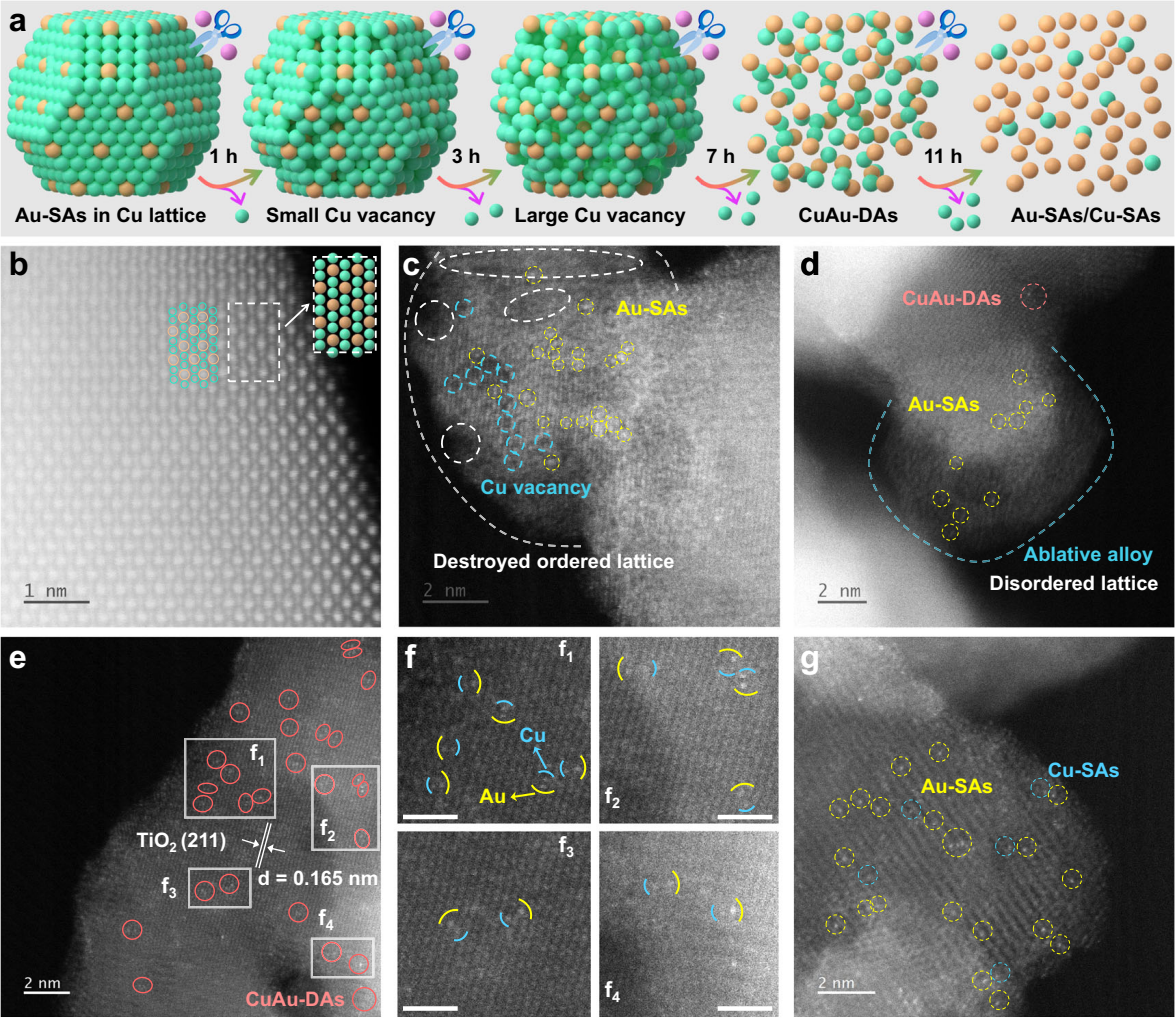
Morphological characterization of the CuAu-based TiO_2_ composites. **a** Dynamic schematic illustration from isolated Au-SAs in the Cu lattice to CuAu-DAs during the vectored etching process (green sphere: Cu^0^; orange sphere: Au^0^; purple sphere: Fe^3+^). AC-HAADF-STEM images of Cu_5_Au_1_-TiO_2_ (**b**), E_1_-Cu_5_Au_1_-TiO_2_ (**c**), E_3_-Cu_5_Au_1_-TiO_2_ (**d**), E_7_-Cu_5_Au_1_-TiO_2_ (**e**), and E_11_-Cu_5_Au_1_-TiO_2_ (**g**). **f** Magnified images of the corresponding areas in image (**e**). Scale bars of **f**_**1**_-**f**_**4**_: 1 nm.

Compared to the widespread bottom-up synthetic strategies of SACs and DACs^32–48,50–58^, the up-bottom ion-cutting architecture controlled by the vectored etching of specific element contents in the alloy may provide novel insight for the adjustable design of SACs and DACs. Figure 1a illustrates the ion-cutting architecture fabrication of Cu nanoclusters (Cu-NCs) decorated Au-SAs (CuAu-NCSAs), CuAu-DAs, and Au-SAs/Cu-SAs supported by TiO_2_ via adjusting the vectored etching time of the Cu_5_Au_1_ alloy. Transmission electron microscopy (TEM) and aberration-correction high-angle annular dark-field scanning transmission electron microscopy (AC-HAADF-STEM) were carried out to acquire a more spatially resolved structural configuration during the different catalyst synthesis processes. In Supplementary Figs. 1 and 2, the commercial TiO_2_ displays the irregular nanoparticle morphology with the ca. 13.6 nm of particle size, and the EDS mapping analysis also verifies the uniform Ti and O elements distribution. Compared to commercial TiO_2_, the regular and larger size of Cu_5_Au_1_ alloy was observed in Supplementary Fig. 3a, and the compactly connected heterointerface was observed between Cu_5_Au_1_ and TiO_2_ (Supplementary Fig. 3b). In addition, the overlapping Cu and Au elements distribution were also exhibited in EDS mapping images, indicating the successful construction of CuAu alloy (Supplementary Fig. 3c–g). As shown in Fig. 1b and Supplementary Fig. 4, the ordered stagger of Cu/Au atoms stacked Cu_5_Au_1_ alloy nanoparticles are successfully loaded on bulk commercial TiO_2_, and the isolated Au single atom in Cu lattice provided the precondition for CuAu-DAs formation. Moreover, the 4.43 and 1.71 Å of Au-Au and Cu-Au atomic distance further verify the isolated Au SAs surrounded by Cu atoms rather than the Au atoms in Cu_5_Au_1_ alloy (Supplementary Fig. 4a–c). After 1 h of vectored etching of Cu atoms (Fig. 1c and Supplementary Fig. 5a–d), slightly destroyed lattice and few Cu vacancies in the Cu_5_Au_1_ alloy were observed due to the destruction of the ordered arrangement of Cu-Au atoms. The ablative Cu_5_Au_1_ alloy and few CuAu-DAs were obtained after 3 h of etching (Fig. 1d and Supplementary Fig. 6a, b), indicating that the constant loss of Cu atoms could promote the collapse of the Cu_5_Au_1_ alloy framework and the redistribution of adjacent Cu-Au atoms on the TiO_2_ surface. Moreover, the distinguished ordered lattice of TiO_2_ and disordered lattice of Cu_5_Au_1_ were simultaneously observed in HRTEM and the corresponding pseudo-color images, which further implied the successfully vectored etching of such Cu_5_Au_1_ alloy (Supplementary Fig. 6c, d). As shown in Fig. 1e, the CuAu-DAs are uniformly dispersed on the surface of the TiO_2_ (211) plane (lattice spacing: 0.165 nm) in E_7_-Cu_5_Au_1_-TiO_2_. In addition, the magnified images (Fig. 1f_1_-f_4_) and acquired AC-HAADF-STEM image intensity profile (Supplementary Fig. 7) clearly verified subnanometer distances (~2–3 Å) between the Cu-SAs (dark) and Au-SAs (bright) according to the different atomic mass of the corresponding elements^59^, suggesting that the twin CuAu-DAs was successfully constructed after 7 h of vectored etching of the Cu_5_Au_1_ alloy. Moreover, when the vectored etching time was prolonged to 11 h (Fig. 1g and Supplementary Fig. 8), a few Cu-SAs and dominated Au-SAs were observed in the AC-HAADF-STEM image, indicating that the dynamic vectored etching process promoted the further dissociation of CuAu-DAs structure due to the consistent decrease of Cu atoms. In Supplementary Fig. 9, Cu-SAs modified TiO_2_ was also constructed by 7 h of vectored etching of Cu-TiO_2_, suggesting the huge potential of such up-bottom ion-cutting technology for atomic-level catalysts design.

**Fig. 2:**
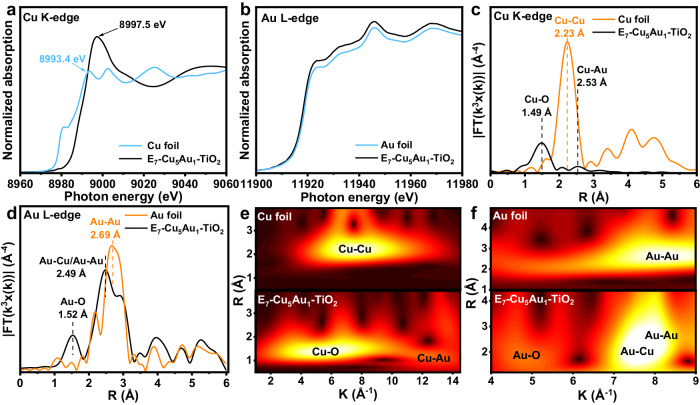
Structural characterization of the CuAu-DAs. XANES analysis of E_7_-Cu_5_Au_1_-TiO_2_ and reference samples at the Cu K-edge (**a**) and Au L-edge (**b**). Corresponding k^3^-weighted FT-EXAFS spectra in the R space for E_7_-Cu_5_Au_1_-TiO_2_ and references at the Cu K-edge (**c**) and Au L-edge (**d**). **e** Cu K-edge WT-EXAFS spectra of Cu foil and E_7_-Cu_5_Au_1_-TiO_2_. **f** Au L-edge WT-EXAFS spectra of Au foil and E_7_-Cu_5_Au_1_-TiO_2_.

To further investigate the atomic-scale configuration of Cu and Au in E_7_-Cu_5_Au_1_-TiO_2_, Cu K-edge and Au L-edge X-ray absorption near-edge structure (XANES) spectroscopy was performed (Fig. 2a, b). Figure 2a illustrates the Cu K-edge XANES spectra for E_7_-Cu_5_Au_1_-TiO_2_ with Cu foil benchmarks as reference, and the absorption edge position of E_7_-Cu_5_Au_1_-TiO_2_ is more positive than that of Cu foil, indicating that partial Cu could directly connect with the lattice O of TiO_2_. In Fig. 2b, the white line peak of E_7_-Cu_5_Au_1_-TiO_2_ exhibits Au characteristic features, which is similar to those of the reference Au foil, indicating the presence of Au^0^. As shown in Fig. 2c, the local coordination around the Cu-O shell and Cu-M shell (Cu-Cu and Cu-Au) was determined by the k^3^-weighted Fourier transform of the extended X-ray absorption fine structure (FT-EXAFS) spectrum. A predominant peak at ~1.49 Å of E_7_-Cu_5_Au_1_-TiO_2_ is assigned to the Cu-O coordination. Furthermore, the other obvious characteristic peak at ~2.53 Å is observed in the E_7_-Cu_5_Au_1_-TiO_2_ spectrum but not in the Cu foil spectrum, implying the possible formation of Cu-Au coordination. The local peak at ~1.52 Å in the Au L-edge spectrum of CuAu-DAs-TiO_2_ (Fig. 2d) is close to that of Au-O, indicating the formation of Au-O coordination. An apparent path at ~2.49 Å is observed in the Au L-edge spectrum of E_7_-Cu_5_Au_1_-TiO_2_ (Fig. 2d), which is close to the values of Cu-Au and Au-Au (Fig. 2c). The Cu foil is used to calculate the standard amplitude reduction factor (*S*_0_^2^ = 0.845, Supplementary Table 2), and the Cu K-edge EXAFS analysis of E_7_-Cu_5_Au_1_-TiO_2_ in R spaces is exhibited in Supplementary Fig. 10. The EXAFS spectrum of E_7_-Cu_5_Au_1_-TiO_2_ is analyzed by using two backscattering paths (Cu-O and Cu-Au). The best-fitting results exhibited that the coordination number of the O and Au in the first coordination sphere of E_7_-Cu_5_Au_1_-TiO_2_ is fitted to be ≈3.3 and ≈1.2 at distances of 1.93 and 2.91 Å, respectively, implying the Cu-SAs is merely adjacent with single Au atom. Therefore, there is no doubt that the existence of CuAu-DAs structure under such vectored etching process, while it is still recognized indeed small existence of pure Au phase due to the shortage of such solvothermal method for the fabrication of highly ordered CuAu alloy. Moreover, the concurrent detection of Cu-M and Au-M distances is in the range of the observed distribution of twin dual atoms in atomic-resolution STEM imaging (Supplementary Fig. 7), further demonstrating that the Cu-Au bond originated from the initial CuAu alloy rather than from Cu-SAs and Au-SAs rearrangement during the etching process. The Cu K-edge and Au L-edge EXAFS oscillations are also analyzed by the wavelet transform (WT) method to further confirm the presence of the Cu-Au path. No WT maxima at 7.5 Å^−1^ (Cu-Cu bond) and 8.3 Å^−1^ (Au-Au bond) were observed in the spectra of E_7_-Cu_5_Au_1_-TiO_2_ (Fig. 2e, f), indicating the SAs-structure configuration of most Cu and Au sites. The WT maxima at 5.7 Å^−1^ and 13 Å^−1^ in the spectra of E_7_-Cu_5_Au_1_-TiO_2_ correspond to the Cu-O and Cu-Au bonds, respectively (Fig. 2e). Similarly, the WT maxima at 4.9 Å^−1^ and 7.7 Å^−1^ are attributed to the Au-O and Au-Cu/Au-Au bonds (Fig. 2f), respectively, consistent with the FT-EXAFS results (Fig. 2c, d). Consequently, the adjacent Cu and Au coexisted as twin diatomic centers and connected with the lattice O of TiO_2_ to form O-Cu-Au-O.

As shown in Supplementary Fig. 11a, the Cu 2p X-ray photoelectron spectroscopy (XPS) binding energy peaks at approximately 932.23 and 952.03 eV are attributed to Cu^0^/Cu^+^^60^. The Auger electron spectrum (Cu LMM) was obtained to further distinguish between Cu^0^ and Cu^+^ in Cu-TiO_2_ and Cu_5_Au_1_-TiO_2_. As shown in Supplementary Fig. 12, the characteristic kinetic energy peaks at 918.06 and 921.82 eV^61^ have corresponded to the electron state of Cu^0^ in Cu-TiO_2_ and Cu_5_Au_1_-TiO_2_. The presence of weak peaks located at 934.70 and 954.40 eV confirms the presence of trace Cu^2+^ in Cu-TiO_2_ and Cu_5_Au_1_-TiO_2_. Notably, no Cu^0^ or Cu^2+^ were detected in E_7_-Cu_5_Au_1_-TiO_2_ because Cu was below the detection limits after the cooperative Fe^3+^ and H^+^ etching reaction of Cu^0^ and Cu^2+^, consistent with the rare residual Cu-SAs observed in AC-HAADF-STEM (Fig. 1e). In Supplementary Fig. 11b, the two Au 4f peaks of Au-TiO_2_ located at 82.99 (Au 4f_7/2_) and 86.70 eV (Au 4f_5/2_) are attributed to zero valence Au. Compared to those of Au-TiO_2_, the Au 4f_7/2_ and Au 4f_5/2_ characteristic peaks of Au_5_Cu_1_-TiO_2_ are positively shifted to 83.18 and 86.90 eV, indicating the impeded electron transfer from TiO_2_ to Au due to the surrounding Cu barrier. After the etching process, the Au 4f binding energy peaks of E_7_-Cu_5_Au_1_-TiO_2_ are more negative than those of Au-TiO_2_ and Cu_5_Au_1_-TiO_2_, illustrating that more TiO_2_ local electrons are transferred to Au due to the decreased amounts of Cu, further suggesting the direct concatenation between Au and Cu. The most negative Au 4f binding energies of E_7_-Cu_5_Au_1_-TiO_2_ suggest the high-concentration electron density of Au, which inevitably benefits the multiple electron reaction conduction on the Au sites. Consequently, large amounts of photogenerated electrons may be transferred from TiO_2_ to the CuAu-DAs due to the low Femi level of metallic Cu and Au^62,63^, which may cause both Cu-SAs and Au-SAs to be the main centers for CO_2_ adsorption-activation and C-C coupling. Significantly, the Ti 2p spectra of E_7_-Cu_5_Au_1_-TiO_2_ exhibits peaks of Ti 2p_3/2_ and Ti 2p_1/2_ at more negative binding energies compared to those of Cu-TiO_2_, Au-TiO_2_, and Cu_5_Au_1_-TiO_2_ (Supplementary Fig. 11c), further indicating that the Cu-SAs and Au-SAs are more beneficial for photogenerated electron migration from TiO_2_ to the CuAu-DAs under irradiation. All the O 1s spectra of the as-prepared samples show two typical peaks, which are assigned to the O-Ti bond of TiO_2_ and the O–H bond of surface adsorbed OH groups^62^ (Supplementary Fig. 11d). X-ray diffraction (XRD) was conducted to further analyze the crystal surface information of these TiO_2_-based samples. Compared to anatase TiO_2_ (JCPDS No. 71-1166), the as-prepared Au-TiO_2_ and Cu_5_Au_1_-TiO_2_ composites gives rise to (200), (220), and (311) characteristic peaks of Au (JCPDS 65-2870), while no characteristic peak of Cu was observed in Cu_5_Au_1_-TiO_2_ and Cu-TiO_2_ (Supplementary Fig. 13), indicating the rapid growth of pure Au phase and the restrained ordered growth of Cu in the CuAu structure due to the different standard electrode potential (*E*^0^) of Au ([AuCl_4_]^-^/Au^0^, *E*^0^ = +0.93 V) and Cu (Cu^2+^/Cu^0^, *E*^0^ = +0.34 V)^64^. Notably, the HRTEM and EDS mapping images exhibited the small-sized Cu nanoparticle (ca. 7.9 nm) decorated TiO_2_ (Supplementary Fig. 14a–d), and the EDS mapping also showed the uniformly dispersed Cu elements (Supplementary Figs. 14 and 15), which indicated that Cu existed as the small-sized scale rather than extended growth into large-sized structure (Supplementary Fig. 14e–h). There has been reported that the low ordering degree of CuAu alloy merely display the Au characteristic peaks due to the dominated Miller indexes of Au phase^65^, which is corresponded to the observation of weak Au characteristic peaks in our work. Although the AC-HAADF-STEM and EDS mapping display the Cu atoms surrounded isolated Au single atoms (Supplementary Fig. 4d–g), the predominated 0.233 nm of lattice spacing was observed in the AC-HAADF-STEM of Cu_5_Au_1_-TiO_2_, which is ascribed to the (111) crystal facet of Au rather than Cu and CuAu characteristic lattice planes. Therefore, the apparent characteristic crystal facet of Au that displayed in AC-HAADF-STEM images and XRD reflections could be ascribed to the dominated Miller indexes of Au phase (Supplementary Figs. 4d and 13). According to the dynamic XRD patterns of Cu_5_Au_1_-TiO_2_ after different vectored etching time (Supplementary Fig. 16a, b), no obvious enhanced Au characteristic peak intensity of E_t_-Cu_5_Au_1_-TiO_2_ was observed compared to that of Cu_5_Au_1_-TiO_2_ after the vectored etching process, suggesting that the vectored etching processes of Cu could not promote isolated Au-SAs rearrangement to form Au lattice plane. Inductively coupled plasma-atomic emission spectroscopy (ICP‒AES) was conducted to evaluate the dynamic Cu/Au molar ratios during the etching process. In Supplementary Fig. 17, with increasing etching time, the Cu/Au molar ratio value of E_t_-Cu_5_Au_1_-TiO_2_ decreases deeply, while the molar ratio values of H_t_-Cu_5_Au_1_-TiO_2_ decrease slightly, indicating the presence of large amounts of Cu^0^ rather than Cu^+^/Cu^2+^ and the successful construction of an adjustable CuAu atomic-level nanostructure. Owing to the different redox capacity-related standard electrode potential between Fe^3+^ (Fe^3+^ + e^-^ → Fe^2+^, *E*^0^ = +0.77 V) and Cu^0^ (Cu^2+^ + 2e^-^ → Cu^0^, *E*^0^ = +0.34 V), the Fe^3+^ could spontaneously be reduced by Cu^0^ (Fe^3+^ + Cu^0^ → Fe^2+^ + Cu^2+^)^64^. However, due to the insufficient oxidized capacity of Fe^3+^ for Au^0^ (Au^3+^ + 3e^-^ → Au^0^, *E*^0^ = +1.52 V) oxidation^64^, the contents of Au keep constant during the whole etching process (Supplementary Table 3), which seriously restrains the dissolution of Au^0^ and promote the redistribution of Au atoms on TiO_2_. Localized surface plasmon resonance (LSPR) is often related to the metal shape and the dielectric constant of the surrounding medium^66^. Therefore, the connection between atomic interface engineering and the light absorption of the as-prepared samples was also checked by UV‒vis‒NIR diffuse reflection spectroscopy (DRS). As shown in Supplementary Fig. 18a, an obvious redshift is observed over metal-decorated TiO_2_ compared to pure TiO_2_, implying that Cu and Au could effectively enhance visible light absorption and produce more photogenerated carriers. In Supplementary Fig. 19a, E_t_-Cu_5_Au_1_-TiO_2_ shows strikingly increased Au (~520 nm) and Cu (~730 nm) LSPR response peaks compared to Cu_5_Au_1_-TiO_2_, indicating that the regularly variational nanoscale of Au and Cu can benefit the formation of higher electron density centers upon irradiation. However, with the persistent etching of Cu, the LSPR response of Cu almost disappeared due to the consistently decreased content of Cu. Moreover, H_t_-Cu_5_Au_1_-TiO_2_ show much weaker Au LSPR response than E_t_-Cu_5_Au_1_-TiO_2_ (Supplementary Fig. 19b, c), and the negligible Cu LSPR response intensity of H_t_-Cu_5_Au_1_-TiO_2_ confirms that the H^+^ etching process eliminated only a small amount of surficial oxidized Cu to a certain extent, which implied that E_t_-Cu_5_Au_1_-TiO_2_ could induce a higher photogenerated carrier density on CuAu sites due to the stronger Cu and Au LSPR response intensity.

To reveal the charge carrier dynamics on CuAu-DAs modified TiO_2_, photoluminescence (PL) spectroscopy and time-resolved photoluminescence (TRPL) spectroscopy were carried out. As shown in Supplementary Fig. 20a, the intensity of the emission peaks of Cu-TiO_2_, Cu_5_Au_1_-TiO_2_, and E_7_-Cu_5_Au_1_-TiO_2_ at ~425 nm decrease considerably compared to that of pure TiO_2_, indicating that the decoration of metallic cocatalysts on TiO_2_ can improve the charge separation and transfer. Notably, the spectrum of E_7_-Cu_5_Au_1_-TiO_2_ displays a weaker emission peak than that of Cu_5_Au_1_-TiO_2_, indicating the lower photogenerated charge recombination of E_7_-Cu_5_Au_1_-TiO_2_ due to the feedthrough charge transfer channel between TiO_2_ and CuAu-DAs. It is reported that the contributions of τ_1_ and τ_2_ are more related to charge transfer, and the PL decay is more dominated by τ_3_^67–69^. In Supplementary Fig. 20b and Table 4, the CuAu-DAs modified TiO_2_ exhibits shorter τ_1_, τ_2_, and τ_3_ compared to TiO_2_, illustrating the compact interaction and suppressed charge recombination between CuAu-DAs and TiO_2_. Moreover, the shortest τ_3_ lifetime of CuAu-DAs modified TiO_2_ represents the fastest decay in CuAu-DAs-TiO_2_, which is ascribed to the fact that the direct connection between CuAu-DAs and TiO_2_ is more beneficial for convenient photogenerated charge transfer from TiO_2_ to CuAu-DAs rather than recombination in the Cu and Cu_5_Au_1_ bulk. Transient photocurrent and electrochemical impedance spectroscopy (EIS) measurements were also conducted to further reveal the efficiency of photogenerated charge separation and transportation of the as-synthesized samples. In Supplementary Fig. 20c, d, the highest photocurrent density and smallest arc radius of E_7_-Cu_5_Au_1_-TiO_2_ suggest that the CuAu-DAs exhibits much higher charge separation efficiency and faster interfacial charge transportation than TiO_2_, Cu-TiO_2_, and Cu_5_Au_1_-TiO_2_. Therefore, the superior photoreduction CO_2_ performance of CuAu-DAs-TiO_2_ could also be attributed to the excellent photoabsorption and high-efficiency charge separation.

### Photocatalytic performance toward CO_2_ photoreduction

**Fig. 3:**
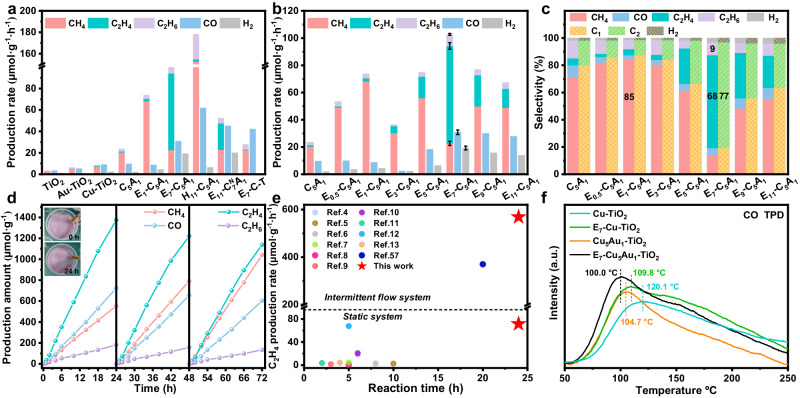
Photocatalytic performance of CuAu-based TiO_2_ composites. CH_4_, C_2_H_4_, C_2_H_6_, CO, and H_2_ production rates of the as-prepared photocatalysts (**a**) and E_t_-Cu_5_Au_1_-TiO_2_ (**b**). **c** Electron-based selectivity of photocatalytic CO_2_ conversion over E_t_-Cu_5_Au_1_-TiO_2_. **d** Long-term photocatalytic stability test of E_7_-Cu_5_Au_1_-TiO_2_. **e** C_2_H_4_ production rate of E_7_-Cu_5_Au_1_-TiO_2_ with the reaction time in comparison with recent reports during the closed glass photoreduction of CO_2_ with a H_2_O gas-circulation system without any sacrificial agents. **f** CO-TPD profile of the as-prepared samples.

A suitable band edge position of TiO_2_ is a prerequisite for the successful photoconversion of CO_2_ gas and H_2_O vapor into CO, CH_4_, C_2_H_4_, C_2_H_6_, and O_2_ upon 320–780 nm irradiation (Supplementary Fig. 18e). To uncover the influence of different CuAu atomic interfaces on the CO_2_ photoreduction reaction, photocatalytic CO_2_ reduction tests were carried out on different CuAu nanostructure-decorated TiO_2_ samples. At the same loading amounts, Cu-TiO_2_ and Au-TiO_2_ achieved much higher CO and CH_4_ production rates than pure TiO_2_ (Fig. 3a and Supplementary Fig. 21a, b) due to the increased number of active sites and the improved charge transfer efficiency^70^. As shown in Fig. 3a and Supplementary Fig. 21a, b, Cu_5_Au_1_-TiO_2_ performs a higher CH_4_ production rate (20.4 µmol·g^−1^·h^−1^) than Cu-TiO_2_ and Au-TiO_2_, which is attributed to the fact that the Au matrix often functions as an electron sink, while the adjacent surficial Cu serves as CO_2_ activation centers and proton transfer stations^70,71^, and the intermediate further combines with the surrounding abundant electrons originating from the Au matrix to promote CH_4_ production through the carbene pathway^62,72,73^. The actual active sites merely exist on the exposed alloy surface rather than in the interior of the bulk, resulting in the catalytic reaction typically occurring on the unsaturated active sites of the CuAu alloy surface. Therefore, photocatalytic CO_2_ reduction tests of the etched samples were also performed to further investigate the influence of CuAu interfacial engineering on the photoreduction of CO_2_. In Supplementary Fig. 22, a possible schematic illustration is shown to describe the dynamic change in the CuAu structural configuration during the constant vectored etching process and define a possible correlation with the photocatalytic performance. With increasing vectored etching time, a peculiar dual volcanic relationship of the hydrocarbon production rate is observed in the E_t_-Cu_5_Au_1_-TiO_2_ series (Fig. 3b). After 1 h of vectored etching of Cu_5_Au_1_-TiO_2_, E_1_-Cu_5_Au_1_-TiO_2_ reaches the highest CH_4_ production (68.2 µmol·g^−1^·h^−1^) and electron-based selectivity (84.7%) among the series of E_t_-Cu_5_Au_1_-TiO_2_ (Fig. 3b and Supplementary Fig. 21c, d), which is ascribed to the increased number of unsaturated Au and Cu active sites (Supplementary Fig. 22). Nevertheless, the CH_4_ production rate continuously decrease due to the constant decrease in the number of Cu atom active sites after a longer vectored etching time. Notably, when the etching time is prolonged to 7 h (Fig. 3b, c and Supplementary Fig. 21c, d), the C_2_H_4_ and C_2_H_6_ production rates of E_7_-Cu_5_Au_1_-TiO_2_ reach 71.6 and 8.5 µmol·g^−1^·h^−1^, respectively, and the electron-based selectivity reached 68.3% (C_2_H_4_) and 9.4% (C_2_H_6_). The dramatically increased C_2_H_4_ production rate of E_7_-Cu_5_Au_1_-TiO_2_ is 305 and 73 times higher than that of TiO_2_ and Cu_5_Au_1_-TiO_2_, respectively, which is ascribed to the superior synergistically enhanced C-C coupling over CuAu-DAs. With the further etching of Cu-SAs, the C_2_H_4_ production rate of E_9_-Cu_5_Au_1_-TiO_2_ and E_11_-Cu_5_Au_1_-TiO_2_ constantly decrease due to the constantly decreasing amounts of Cu-SAs, implying that the mere presence of Au-SAs (Fig. 1g) could not satisfy the high-efficiency C-C coupling and C_2_H_4_ production. Moreover, the optimized amount of E_7_-Cu_5_Au_1_-TiO_2_ photocatalysts was verified to be 20 mg with a highest C_2_H_4_ production rate based on the excellent mass transfer and superior light utilization (Supplementary Fig. 23), and the apparent decrease of C-based products production rate was observed with the addition of superfluous photocatalysts, which could be ascribed to the impeded light transmission^74^. Compared to Au-SAs modified TiO_2_, Cu-SAs (Supplementary Fig. 9) modified TiO_2_ (E_7_-Cu-TiO_2_) exhibits dramatically increased CO and CH_4_ generation, while negligible C_2_ generation is also observed on E_7_-Cu-TiO_2_, indicating that the Cu-SAs is beneficial for high-efficiency *CO production (Supplementary Fig. 24), consistent with recent research results on Cu-SAs based photocatalytic CO_2_ reduction^75–79^. Therefore, we suspect that only the specific existence of adjacent Cu-SAs and Au-SAs can efficiently and synergistically favor C-C coupling and C_2_H_4_ production. To further confirm that the CuAu-DAs structure can promote C_2_H_4_ generation, a photoreduction CO_2_ test was also conducted on E_t_-${{{\mathrm{Cu}}}}_{7}^{{{\rm{N}}}}$Au_1_-TiO_2_ (Supplementary Fig. 25) constructed by adding additional Cu content to the Cu_5_Au_1_ alloy. The dual volcanic relationship of the hydrocarbon production rate is still observed on E_t_-${{{\mathrm{Cu}}}}_{7}^{{{\rm{N}}}}$Au_1_-TiO_2_ based on the transformation of the CuAu structure. Furthermore, E_11_-${{{\mathrm{Cu}}}}_{7}^{{{\rm{N}}}}$Au_1_-TiO_2_ exhibits the maximum C_2_H_4_ production rate after 11 h of vectored etching (Supplementary Fig. 25a), and the CO production also rapidly increase with enhanced C_2_H_4_ generation, similar to the behavior of E_7_-Cu_5_Au_1_-TiO_2_, indicating that the *CO species is a critical intermediate for C-C coupling, as reported^6,14,53^. As shown in the ICP‒AES results (Supplementary Fig. 26 and Table 5), the Cu/Au molar ratios of both E_7_-Cu_5_Au_1_-TiO_2_ and E_11_-${{{\mathrm{Cu}}}}_{7}^{{{\rm{N}}}}$Au_1_-TiO_2_ are similar, further confirming that the dominated CuAu DAs-structure dramatically actuate C_2_H_4_ production. Moreover, photoreduction CO_2_ tests were also conducted on H_t_-Cu_5_Au_1_-TiO_2_ to further identify the effects of different CuAu structures. Although a superior CO production rate (61.8 µmol·g^−1^·h^−1^) is observed on H_11_-Cu_5_Au_1_-TiO_2_ (Supplementary Fig. 27), the C_2_ production is still nonideal, emphasizing that only Au-SAs, rather than low-coordination Au sites, could rapidly convert the high-concentration CO into C_2_ products. Furthermore, a superhigh CH_4_ (152.6 µmol·g^−1^·h^−1^) production rate is observed for H_11_-Cu_5_Au_1_-TiO_2_, indicating that low-coordination Au sites in the Cu lattice are more beneficial for converting *CO into CH_4_ than *CO coupling on Au-SAs. Consequently, in such CuAu-DAs modified TiO_2_ photocatalytic systems, the Cu-SAs guarantee rapid *CO generation and high-concentration coverage, and the adjacent Au-SAs further promote migration and coupling of the generated *CO. The source of photoreduced CO_2_ products was investigated by using isotope labeling ^13^CO_2_ and H_2_^18^O as the reactant under irradiation, and the products were analyzed by gas chromatography‒mass spectrometry (GC‒MS). The GC‒MS peak sequences of ^13^CO, ^13^CH_4_, ^13^C_2_H_4_, and ^13^C_2_H_6_ are shown in Supplementary Fig. 28, and the peaks at *m*/*z* = 29, *m*/*z* = 17, *m*/*z* = 30, and *m*/*z* = 32 are assigned to ^13^CO, ^13^CH_4_, ^13^C_2_H_4_, and ^13^C_2_H_6_, indicating that the carbon source of CO and hydrocarbons is indeed derived from the input CO_2_ gas. Overall CO_2_ photoreduction is divided into two major half reaction steps, the CO_2_ reduction and H_2_O oxidation^12,80^, and the detection of ^16^O^18^O and ^18^O_2_ species verifies that O_2_ originates from H_2_O oxidation in the photocatalytic CO_2_ reduction in (Supplementary Fig. 28). Notably, in Supplementary Fig. 29, O_2_ evolution related to holes consumption of the TiO_2_ based composites are also stoichiometrically approximate to products of photogenerated electrons reduction, which indicates the simultaneous CO_2_ reduction and H_2_O oxidation behaviors. The CO_2_ photoreduction experiment was also taken under no existence of H_2_O to further figure out the influence of H_2_O species (Supplementary Fig. 30). No C-based product was detected in the absence of H_2_O, indicating the significance of photogenerated holes consumption in the overall CO_2_ photoreduction. Therefore, both the investigation of H_2_O oxidation and CO_2_ reduction are crucial and directive for the development of photocatalysis. Furthermore, a negligible amount of CO production was detected on E_7_-Cu_5_Au_1_-TiO_2_ and E_7_-Cu_5_Au_1_-Al_2_O_3_ upon 420 nm irradiation (Supplementary Fig. 31a), indicating the inappreciable CuAu LSPR effect and the dominant TiO_2_ electron donator in photocatalytic CO_2_ reduction (Supplementary Fig. 31b). Consequently, the same DAs-modified strategy was conducted on the widespread reported photocatalysts of carbon nitride (C_3_N_4_) to further verify the universality of such optimized strategy, and the vectored etching Cu_5_Au_1_ modified C_3_N_4_ and TiO_2_ also exhibit the efficient C_2_H_4_ production, which sufficiently prove the universality of such CuAu-DAs modification for the optimization of C-C coupling reaction (Supplementary Figs. 32–35).

Cycling tests were performed to analyze the relationship between activity and stability during the photoreduction of CO_2_ into C_2_H_4_ (Fig. 3d). Based on the limitation of catalyst stability, the majority of photocatalytic CO_2_ reduction into C_2_H_4_ are maintained for only a few hours and accompany with C_2_H_4_ production stagflation due to the poor structural stability and surface poisoning effect on the photocatalyst (Fig. 3e)^4–13,57^. As shown in Fig. 3e, regardless of the low-efficiency or high-efficiency C_2_H_4_ production rate on different photocatalysts, all exhibited limited reaction time due to the deactivation of photocatalysts. Interestingly, in this work, the yield of each product increases linearly during three cycles of 72 h irradiation, as shown in Fig. 3d, and the AC-HAADF-STEM image of E_7_-Cu_5_Au_1_-TiO_2_ displays an unchanged CuAu-DAs structural configuration, as shown in Supplementary Fig. 36, indicating the superior structural stability of E_7_-Cu_5_Au_1_-TiO_2_ under such high-efficiency C_2_H_4_ production in the static system. Moreover, compared to the traditional static system, the intermittent flow system is considered a better choice for the improvement of photocatalytic CO_2_ reduction due to the high-efficiency transport of mass^81–86^. Therefore, the intermittent flow system is also adopted to further analyze the stability of photocatalysts under higher efficiency CO_2_ conversion circumstances. Notably, in such an intermittent flow system, the C_2_H_4_ production rate reaches 568.8 µmol·g^−1^·h^−1^ (Supplementary Fig. 37a) after 24 h of irradiation, which is superior to those reported in recent works (Fig. 3e). After 5 days of irradiation, C_2_H_4_ production maintains a rate of 483.2 µmol·g^−1^·h^−1^, indicating the superhigh stability of CuAu-DAs catalysts even during such high-efficiency C_2_H_4_ conversion. In addition, a series of long-term photocatalytic stability tests were carried out on different metallic structure-modified TiO_2_ to determine the reason for the superior stability of such specific CuAu-DAs, and CO temperature-programmed desorption (CO-TPD) was also conducted to further analyze the connection between the catalyst structure and CO affinity. As shown in Fig. 3f, Cu-TiO_2_ shows the strongest CO adsorption strength centered at ~120.1 °C in the weak absorption area compared to other catalysts^7^, indicating that CO desorption is most difficult on this catalyst surface, which may suppress fresh CO_2_ adsorption and conversion on Cu-TiO_2_ during the photocatalytic CO_2_ reduction process. As shown in Supplementary Fig. 38, Cu-TiO_2_ also exhibits nonlinear CO generation, which is ascribed to the partially deactivated Cu sites induced by strong CO absorption. Furthermore, an apparent color change (yellow to black) of Cu-TiO_2_ after 24 h of photocatalytic CO_2_ reduction is noticed (Supplementary Fig. 38), and the black samples became yellow after 100 °C annealing in a vacuum-treated system (Supplementary Fig. 39), further indicating that the color change could be induced by intermediate adsorption during photocatalytic CO_2_ reduction. Furthermore, the possible absorbed isotopically labeled ^13^C intermediates of Cu-TiO_2_ after 24 h photocatalytic CO_2_ reduction and subsequent 100 °C annealing in a vacuum-treated system were analyzed by GC‒MS. The GC‒MS peak sequences of ^13^CO are shown in Supplementary Fig. 40, and the peak at *m*/*z* = 29 is assigned to ^13^CO, indicating the occurrence of CO poisoning on Cu-TiO_2_, consistent with the results of the CO-TPD analysis and photocatalytic stability test. However, the CO adsorption strength (109.8 °C) of E_7_-Cu-TiO_2_ is obviously lower than that of Cu-TiO_2_ (Fig. 3f), indicating that the contractible Cu size (nanoparticle to single atom) partially alleviated the CO poisoning effect. Although the CO production of E_7_-Cu-TiO_2_ increases drastically compared to that of Cu-TiO_2_ due to the decreased Cu coordination number, E_7_-Cu-TiO_2_ still exhibits nonlinear CO generation and a slight color change (white to pale yellow, Supplementary Fig. 41) due to its slight surface CO poisoning (Supplementary Fig. 42), implying that low-coordination-number Cu inevitably suffers CO poisoning. Notably, the CO adsorption strength of Cu_5_Au_1_-TiO_2_ is lower than that of Cu-TiO_2_ (Fig. 3f), confirming that the introduction of Au in the Cu lattice weakens the CO adsorption of Cu, as reported^53^. The linearly increased CH_4_ yield (Supplementary Fig. 43) and weakened CO adsorption (Fig. 3f) of Cu_5_Au_1_-TiO_2_ compared to Cu-TiO_2_ suggest that the isolated Au in the Cu lattice efficiently promote *CO conversion to CH_4_ on the Au sites and suppressed *CO accumulation, which benefits the resistance to catalyst deactivation. However, Cu_5_Au_1_-TiO_2_ also displays a nonlinearly increased CO yield induced by weak CO poisoning due to the large amount of Cu in the Cu_5_Au_1_ alloy (Supplementary Figs. 43 and 44), which further implies that the addition of Au not only weakens CO adsorption on Cu sites but also serves as a *CO turnover site to rapidly consume the *CO arising from Cu to alleviate the CO poisoning effect on Cu sites. Moreover, the CO-TPD analysis indicates that E_7_-Cu_5_Au_1_-TiO_2_ exhibits the highest CO adsorption capacity and lowest CO desorption temperature at ~100.0 °C, as shown in Fig. 3f, implying the enormous capacity for CO coverage and the most resistant CO poisoning on CuAu-DAs, which is beneficial for the high efficiency and stability of *CO coupling. Based on the weakest CO chemical adsorption strength of E_7_-Cu_5_Au_1_-TiO_2_, no ^13^CO of E_7_-Cu_5_Au_1_-TiO_2_ was detected by GC‒MS after a 24 h stability test and 100 °C annealing in a vacuum-treated system (Supplementary Fig. 45). Meanwhile, there is also no special characteristic peaks of C-based residual surficial absorbates was observed in FTIR spectrum after CO_2_ photoreduction over E_7_-Cu_5_Au_1_-TiO_2_ (Supplementary Fig. 46), suggesting the inexistence of residual C-based intermediate and the superior stability of such CuAu-DAs structure. Owing to the synergistic effect of CuAu-DAs heteronuclear sites, rapid *CO coupling and weakened CO adsorption are simultaneously realized, ensuring superior catalytic sustainability even under such high-efficiency C_2_H_4_ production.

### Mechanism of the photocatalytic performance

**Fig. 4:**
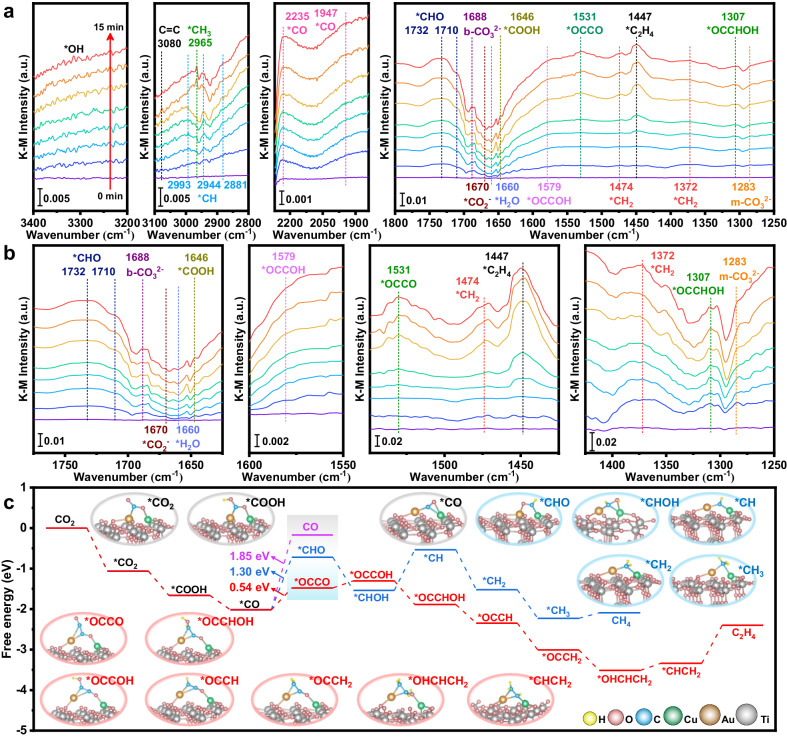
Schematics of C_2_H_4_ generation during photocatalytic CO_2_ reduction. **a** In situ DRIFTS detection of E_7_-Cu_5_Au_1_-TiO_2_. **b** Corresponding magnifying area of the in situ DRIFTS spectra in **a**. **c** The free energy diagram of CO_2_ conversion over the CuAu-DAs-TiO_2_ photocatalyst, together with the atomic structures of the reaction intermediates.

Time-dependent in situ diffuse reflectance Fourier transform infrared spectroscopy (DRIFTS) was employed to elucidate the reaction intermediates and concrete evidence of the reaction mechanism under 355 nm laser irradiation for 15 min (Fig. 4a, b). Humid CO_2_ was carried into the reaction chamber until equilibrium was reached, and different infrared adsorption characteristic peaks of E_7_-Cu_5_Au_1_-TiO_2_ were gradually observed when the photocatalyst was subjected to constant irradiation. As shown in Fig. 4b, monodentate carbonate (m-CO_3_^2-^ at 1283 cm^−1^) and bidentate carbonate (b-CO_3_^2-^ at 1688 cm^−1^) are generated from the co-adsorption of CO_2_ and H_2_O on the surface of E_7_-Cu_5_Au_1_-TiO_2_^57,87–90^. The peaks at 1670 and 1646 cm^−1^ are attributed to the vibrations of *CO_2_^−^ and *COOH groups^7,91,92^, respectively. Moreover, the characteristic peak at 1660 cm^−1^ is assigned to H_2_O decomposition signals^93^, and the constantly increased broad IR bands at 3200–3400 cm^−1^ are corresponded to the vibration of *OH groups generated from water dissociation under simulated irradiation^4,6,94^. The different characteristic peaks at approximately 1947 and 2235 cm^−1^ are assigned to *CO intermediates^4,7,95^, including Cu-CO and Au-CO on E_7_-Cu_5_Au_1_-TiO_2_ (Fig. 4a). Moreover, the contact angles of Cu_5_Au_1_-TiO_2_ and E_7_-Cu_5_Au_1_-TiO_2_ are 9° and 7° (Supplementary Fig. 47), respectively, indicating the better surface hydrophilicity of E_7_-Cu_5_Au_1_-TiO_2_, which further demonstrates that the low-coordinated Cu and Au atoms are beneficial for H_2_O adsorption and the generation of protons to further facilitate the protonation reaction. Coincidently, asymmetric vibration of *OCCO is observed at ~1531 cm^−1^ (Fig. 4b), providing significant evidence for the C_2_ evolution pathway, indicating that the C_2_ products arise from the coupling of *CO intermediates^4,94,96,97^. Moreover, the unique C-C coupling intermediates *C = C (3080 cm^−1^)^96^, *OCCOH (1579 cm^−1^)^6^, *OCCHOH (1307 cm^−1^)^4^, and *C_2_H_4_ (1447 cm^−1^)^63^ were also detected spectroscopically, further indicating the complexly cascaded multiple electron and proton reaction for the ultimate C_2_H_4_ formation. The vibration frequency of surface-bound *CHO is observed at approximately 1732 and 1710 cm^−1^ on E_7_-Cu_5_Au_1_-TiO_2_ due to the one-electron and one-proton reduced reaction of the *CO intermediate^63,96,98^. Additionally, the peaks at approximately 2993, 2944, and 2881 cm^−1^ are attributed to the C–H symmetric stretching vibrations of methylene (Fig. 4a), facilitating the evolution of CH_4_ or C_2_H_4_ products^4^. The characteristic spectral peaks at approximately 1372, 1474, and 2965 cm^−1^ are attributed to *CH_2_, *CH_2_, and *CH_3_ intermediates, respectively^57,99^, demonstrating that these intermediates could be the source of hydrocarbons. Gibbs free energy theoretical calculations were conducted to elucidate the correlation between the specific nanostructure, electronic properties, and catalytic performance. By combining these results with the time-dependent in situ DRIFTS results, these possible reaction pathways were further proposed by Gibbs free energy calculations. In Fig. 4c, the formations of *CO_2_, *COOH, and *CO on E_7_-Cu_5_Au_1_-TiO_2_ are constantly exothermic and spontaneous processes, indicating that the synergistic effect between Cu-SAs and Au-SAs could significantly promote CO_2_ adsorption and activation to produce large amounts of *CO intermediates, which is beneficial for *CO coupling under such high concentrations of *CO. The hydrogenation and desorption of *CO to *CHO and CO species require 1.30 and 1.85 eV of energy expenditure, respectively, while *CO coupling to *OCCO consume only 0.54 eV of energy input. Because the energy barrier of *CO coupling is much lower than that of *CO hydrogenation and desorption, the *OCCO intermediates are confirmed to form preferentially during the *CO transformation process. Furthermore, under the constant hydrogenation process of *OCCO, the C_2_H_4_ formation paths are theoretically proposed as described in the following formulas: *OCCO ⟶ *OCCOH ⟶ *OCCHOH ⟶ *OCCH ⟶ *OCCH_2_ ⟶ *OHCHCH_2_ ⟶ *CHCH_2_ ⟶ C_2_H_4_. Therefore, the reaction mechanism of photoreduction CO_2_ into C_2_H_4_ is determined in detail on the basis of the time-dependent in situ DRIFTS experiments and density functional theory (DFT) simulations.

### Mechanism for the resistance of catalyst deactivation

**Fig. 5:**
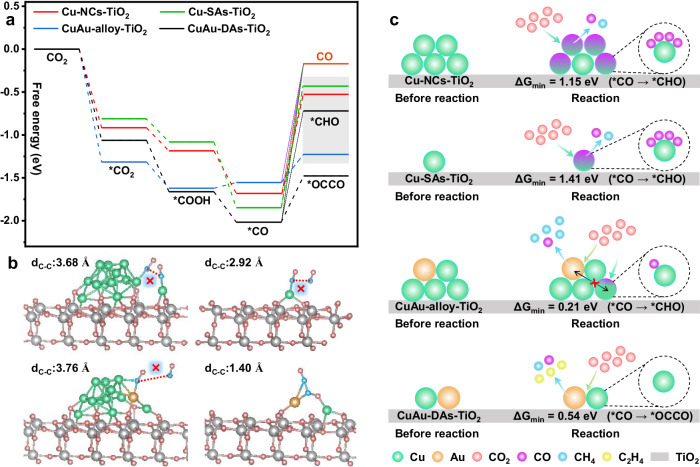
Illustration of the model mechanisms. **a** The free energy diagram of CO_2_ reduction to CO, *CHO, and *OCCO over different modeled surfaces. **b** The most energetically favorable *OCCO adsorption configurations. **c** Schematic illustration of the CO poisoning pathway during the photocatalytic CO_2_ reduction reaction on different models.

The enhanced CO poisoning resistance of CuAu hybrid catalysts during photocatalytic CO_2_ reduction tests compared to that of pure Cu was considered. DFT calculations of Cu-NCs, Cu-SAs, CuAu alloy, and CuAu-DAs modified TiO_2_ models were performed to further analyze the relation between C-based intermediate conversion efficiency and different nanostructures (Fig. 5a). During the *CO generation process, there is almost no energy input for *CO_2_, *COOH, and *CO formation in these models (Fig. 5a), which implies rapid *CO formation due to these exothermic and spontaneous processes. Nevertheless, all the conversions from *CO to *CHO, *OCCO, and CO molecules in these models must overcome an enormous energy barrier, indicating the rate-determined significance of *CO conversion during the CO_2_ reduction process. During three possible *CO conversion routes (*CO ⟶ CO, *CO ⟶ *CHO, *CO ⟶ *OCCO), the optimized energetically favorable *CO coupling modes could not be formed on Cu-NCs-TiO_2_ (C-C distance, 3.68 Å), Cu-SAs-TiO_2_ (C-C distance, 2.92 Å), and CuAu-alloy-TiO_2_ (C-C distance, 3.76 Å) due to the weak interaction between adjacent absorbed *CO intermediates (Fig. 5b). In Supplementary Fig. 48, CuAu-DAs-TiO_2_ (−1.98 eV) presents the highest *CO adsorption energies compared to CuAu-alloy-TiO_2_ (−1.44 eV), Cu-NCs-TiO_2_ (−1.55 eV), and Cu-SAs-TiO_2_ (−1.79 eV), ensuring the compact *CO interaction and high-concentration *CO coverage^100^ (Fig. 5b, C-C distance, 1.40 Å), which contributes to the successful construction of the *OCCO intermediate on CuAu-DAs-TiO_2_ due to the optimized surface adsorption configurations resulting from the cooperative modification of the steric and electronic properties of CuAu-DAs^26,28–31^. Compared to *CO desorption to CO, *CO is preferentially protonated due to the lower energy barrier and energy input for *CHO production on Cu-NCs-TiO_2_ (1.15 eV), Cu-SAs-TiO_2_ (1.41 eV), and CuAu-alloy-TiO_2_ (0.21 eV). Although the transformation of reaction routes could be an efficient way to resist *CO species accumulation, the energy input for *CO conversion to *CHO on Cu-NCs-TiO_2_ and Cu-SAs-TiO_2_ was still enormous, which also dramatically limited the rapid *CO transformation and led to surface *CO accumulation. Affected by the introduction of Au, the energy input of *CO conversion sharply decreased due to the synergistic cooperation between Cu and Au sites, suggesting that the suppressed *CO poisoning effect could be ascribed to the rapid *CO conversion on CuAu sites^101,102^. Moreover, the energy input for *CO conversion on CuAu-DAs-TiO_2_ (*CO to *OCCO, 0.54 eV) was much lower than that on Cu-SAs-TiO_2_ (*CO to *CHO, 1.41 eV) and Cu-NCs-TiO_2_ (*CO to *CHO, 1.15 eV), which suggested that the extremely low C-C coupling energy barrier-induced rapid *CO consumption could be the key factor for the sharp *CO poisoning resistance on CuAu-DAs-TiO_2_ (Fig. 5a, c). Owing to the synergistic cooperation between the adjacent Cu-SAs and Au-SAs, simultaneous and high-efficiency *CO formation and *CO conversion could be realized based on the reconstituted surface reactant intermediate adsorption configurations and reduced *OCCO coupling reaction energy barrier, which efficaciously overcomes the activity-stability seesaw effect in photoreduction of CO_2_ with H_2_O into C_2_H_4_ (Fig. 5c).

## Discussion

In summary, CuAu-DAs modified commercial TiO_2_ was successfully synthesized by an up-bottom atomic synthetic process involving the vectored etching of Cu atoms in a CuAu alloy. According to the activity tests, CO-TPD, and DFT calculation results of the CuAu-DAs structure, Cu-SAs are beneficial for high-efficiency *CO production, while Au-SAs not only moderate the *CO binding strength of the composites but also strikingly increase the *CO coupling efficiency and suppress catalyst deactivation under high-concentration *CO conditions. Owing to such synergistically catalytic effect of heteronuclear DAs, CuAu-DAs-TiO_2_ exhibited a superhigh rate of 568.8 μmol·g^−1^·h^−1^ in an intermittent flow system, and the negligible CO poisoning of CuAu-DAs-TiO_2_ was observed during the 120-h photocatalytic stability test. Herein, our discovery not only provides a novel insight into the adjustable synthesis of atomic-level catalysts but also provides a new technique to optimize the selectivity, activity, and stability of photocatalysts based on reconstituted surface adsorption configurations of reactant intermediates and reduced reaction barriers.

## Methods

### Synthesis of Cu_5_Au_1_-TiO_2_

Two hundred milligrams of commercial TiO_2_, 105 mg of PVP, 120 mg of L-ascorbic acid, and 300 mg of KBr were dispersed in 8 mL of deionized water, sonicated for 10 min in a 50 mL round-bottom flask, and transferred into an 80 °C oil bath under constant stirring. After 10 minutes of heating, 0.083 mmol of CuCl_2_·2H_2_O and 0.017 mmol of HAuCl_4_·4H_2_O were dissolved in 4 mL of H_2_O, and the mixed CuCl_2_·2H_2_O and HAuCl_4_·4H_2_O solution was rapidly transferred into the round-bottom flask. Finally, the solution was kept at 80 °C for 3 h. The sample was washed with deionized water three times and absolute ethanol three times to remove impurities. The obtained samples were denoted as Cu_5_Au_1_-TiO_2_, which could also be simplified to C_5_A_1_.

### Synthesis of E_t_-Cu_5_Au_1_-TiO_2_

FeCl_3_·6H_2_O (0.06 mmol) was dispersed in 10 mL of 0.1 M HCl, and gaseous Ar was bubbled through the solution for 60 min to eliminate dissolved O_2_. Subsequently, 100 mg of Cu_5_Au_1_-TiO_2_ was dissolved into the as-prepared solution. The suspension solution was reacted in an oil bath at 50 °C for different etching reaction time (0.5, 1, 3, 5, 7, 9, and 11 h). The as-prepared samples were washed with 0.1 M HCl and deionized water to remove impurities. The obtained samples were denoted as E_t_-Cu_5_Au_1_-TiO_2_ (simplified as E_t_-C_5_A_1_), where t = 0.5, 1, 3, 5, 7, 9, and 11 corresponded to the etching time.

### Synthesis of E_t_-${{{\mathbf{Cu}}}}_{{{\mathbf{7}}}}^{{{\mathbf{N}}}}$Au_1_-TiO_2_

E_t_-${{{\mathrm{Cu}}}}_{7}^{{{\rm{N}}}}$Au_1_-TiO_2_ was prepared by the same approach as E_t_-Cu_5_Au_1_-TiO_2_, except that the amounts of CuCl_2_·2H_2_O and FeCl_3_·6H_2_O were 0.119 mmol and 0.084 mmol, respectively. The obtained samples were denoted as E_t_-${{{\mathrm{Cu}}}}_{7}^{{{\rm{N}}}}$Au_1_-TiO_2_ (simplified as E_t_-${{{\mathrm{C}}}}_{7}^{{{\rm{N}}}}$A_1_), where t = 0.5, 1, 3, 5, 7, 9, 11, and 13 correspond to the etching time.

### Synthesis of H_t_-Cu_5_Au_1_-TiO_2_

H_t_-Cu_5_Au_1_-TiO_2_ was prepared by the same approach as E_t_-Cu_5_Au_1_-TiO_2_ without the addition of FeCl_3_·6H_2_O. The obtained samples were denoted as H_t_-Cu_5_Au_1_-TiO_2_ (simplified as H_t_-C_5_A_1_), where t = 0.5, 1, 3, 5, 7, 9, 11, and 13 correspond to the etching time.

### Synthesis of Cu-TiO_2_

Cu-TiO_2_ was prepared by the same approach as Cu_5_Au_1_-TiO_2_ without the addition of HAuCl_4_·4H_2_O, and the amount of CuCl_2_·2H_2_O was 0.1 mmol. The obtained sample was denoted as Cu-TiO_2_ (C-T).

### Synthesis of E_t_-Cu-TiO_2_

E_t_-Cu-TiO_2_ was prepared by the same approach as E_t_-Cu_5_Au_1_-TiO_2_. The obtained samples were denoted as E_t_-Cu-TiO_2_ (simplified as E_t_-C-T), where t = 0.5, 1, 3, 5, 7, 9, and 11 correspond to the etching time.

### Synthesis of Au-TiO_2_

Au-TiO_2_ was prepared by the same approach as Cu_5_Au_1_-TiO_2_ without the addition of CuCl_2_·2H_2_O, and the amount of HAuCl_4_·4H_2_O was 0.1 mmol. The obtained sample was denoted as Au-TiO_2_ (simplified as A-T).

### Synthesis of E_7_-Cu_5_Au_1_-Al_2_O_3_

Cu_5_Au_1_-Al_2_O_3_ was prepared by the same approach as E_7_-Cu_5_Au_1_-TiO_2_. The obtained sample was denoted as E_7_-Cu_5_Au_1_-Al_2_O_3_.

### Synthesis of C_3_N_4_

C_3_N_4_ was prepared by polymerization method. 10 g of melamine powder was dispersed into 50 mL of deionized aqueous solution and kept in an ultrasonic bath for 30 min. The mixture was stirred in water bath at 50 °C, and then dried in oven at 60 °C. The as-prepared powder was grounded, and then transferred into ceramic crucible calcined at 300 °C for 1 h, 400 °C for 1 h, and 550 °C for 4 h with 2.5 °C·min^−1^ heating rate in a muffle furnace. After natural cooling to room temperature, the samples were washed by DI water for three times to remove impurity.

### Synthesis of Cu_5_Au_1_-C_3_N_4_

Cu_5_Au_1_-C_3_N_4_ was prepared by the same approach as Cu_5_Au_1_-TiO_2_, except that the TiO_2_ was replaced by C_3_N_4_.

### Synthesis of E_t_-Cu_5_Au_1_-C_3_N_4_

E_t_**-**Cu_5_Au_1_-C_3_N_4_ was prepared by the same approach as E_t_-Cu_5_Au_1_-TiO_2_, except that the Cu_5_Au_1_-TiO_2_ was replaced by Cu_5_Au_1_-C_3_N_4_.

### Characterizations

TEM and EDS mapping analysis (Talos F200X G2 200KV) were applied to confirm the morphologies of the materials. AC-HAADF-STEM was conducted on Titan Cubed Themis G2300 and JEM-ARM200F. XAFS of the Cu K-edge and Au L-edge were measured at the BL14W1 station of the Shanghai Synchrotron Radiation Facility (SSRF, China), and Cu foil and Au foil were used as the reference samples. XAFS data were obtained by means of Athena and Artemis software according to standard procedures. ICP‒AES was carried out on a Varian VISTA-MPX instrument. The crystal structure was characterized by powder XRD using Cu Kα radiation (*λ* = 0.15406 nm) on a Bruker D8 Advance X-ray diffractometer. The compositions of the catalysts were analyzed by XPS (Thermo Scientific K-Alpha). UV‒vis‒NIR DRS was performed on a Shimadzu UV-2450 spectrophotometer. PL and TRPL analyses were conducted by means of an Edinburgh Instruments instrument (FLS-980). TPD tests were performed on an AutoChem1 II 2920 with a thermal conductivity detector. The surface hydrophilicity of the as-prepared samples was determined by contact angle measurement (CA, KSV CM200, Finland).

### Photoelectrochemical tests

Photocurrent and EIS tests were conducted by a CHI660B electrochemical analyzer with a standard three-electrode system, where Ag/AgCl and Pt wire were used as reference and counter electrodes, respectively, and the samples spin-coated onto fluorine-doped tin oxide (FTO) glasses served as working electrodes. During the photocurrent and EIS tests, 0.2 mol·L^‒1^ Na_2_SO_4_ solution and 0.1 mol·L^‒1^ KCl solution containing 5 mmol·L^−1^ K_4_[Fe(CN)_6_]/K_3_[Fe(CN)_6_] were applied as electrolytes. Moreover, the Mott-Schottky test was carried out in a 0.5 mol·L^‒1^ Na_2_SO_4_ solution.

### Photocatalytic activity tests in different reaction systems

(a) Static system: 20 mg of photocatalyst and 5 mL of deionized water were dispersed on a 45 mm diameter quartz glass under sonication and then placed at 60 °C in a vacuum oven for 3 h to dry. Photoreduction CO_2_ reaction tests were conducted on a closed glass gas-circulation system (Labsolar-6A, Beijing Perfectlight Technology Co., Ltd) with the addition of 2 mL deionized water, and a quartz tray was used to separate water and the quartz glass coated by photocatalysts. The temperature was adjusted to 25 °C using circulating water. (b) intermittent flow system: 2.0 mg of photocatalyst was dispersed on the surface of the microporous membrane with a radius of 2.35 cm by filtration and then sealed in the intermittent flow system with the addition of 0.5 mL deionized water. Before irradiation, the gas-circulation system was vacuum-treated for 15 min and then filled with high-purity CO_2_ (99.99%). The reactor filled with high-purity CO_2_ was vacuum-treated again and then filled with high-purity CO_2_ to reach 90 kPa. The photocatalyst was placed 10 cm away from a 300 W Xe lamp (Microsolar 300, 320–780 nm, 250 mW·cm^−2^, Beijing Perfectlight Technology Co., Ltd). Gas products were detected by means of a gas chromatograph (GC9790II, FULI INSTRUMENTS) equipped with a flame ionization detector (FID) and thermal conductivity detector (TCD). The produced gases were calibrated with a standard gas mixture, and the identity was determined by the retention time. ^13^CO_2_ isotope labeling experiments were conducted under the same conditions.

### ^13^CO_2_ isotope labeling experiments

The gas products of ^13^CO_2_ isotope labeling experiments were conducted on Agilent 7890B-5977B GC with MS detector. The HP–5MS type column was adopted to analyze the C-based products. The heating programming was started at 40 °C and maintained for 5 min. The temperature of system was increasing from 40 °C to 290 °C with a heating rate of 20 °C·min^−1^, and the system kept 290 °C for 10 min. Helium was used as a carrier gas in GC with a flow rate of 1 mL·min^−1^. The temperature of gas sampling valve is 280 °C under non-diversion mode. Electron impact ionization with 70 eV voltage under the full scan of 2-350 u. The matching degree of CO, CH_4_, C_2_H_4_, and C_2_H_6_ relied on the database are about 93, 96, 92, and 93%, respectively, which could be regarded as the reference for source of C-based products.

### In situ DRIFTS experiments

In situ DRIFTS experiments were conducted on a Nicolet iS10 (Thermo) machine, and the photocatalysts were sealed in the reaction chamber with a quartz window. CO_2_ and H_2_O were carried into the reaction chamber by N_2_ flow until equilibrium. After taking the equilibrium system before reaction as the blank background, IR signals were collected under 355 nm laser irradiation (3W) through the quartz glass window.

### XAFS date analysis

Data reduction, data analysis, and EXAFS fitting were performed and analyzed with the Athena and Artemis programs of the Demeter data analysis packages^103^ that utilizes the FEFF6 program^104^ to fit the EXAFS data. The energy calibration of the sample was conducted through a standard Cu foil, which as a reference was simultaneously measured. A linear function was subtracted from the pre-edge region, then the edge jump was normalized using Athena software. The k^3^-weighted χ(k) data were Fourier transformed after applying a Kaiser-Bessel window function (Δ*k* = 1.0). For EXAFS modeling, the global amplitude EXAFS (*CN, R, σ*^2^ and Δ*E*_0_) were obtained by nonlinear fitting, with least-squares refinement, of the EXAFS equation to the Fourier-transformed data in R-space, using Artemis software, EXAFS of the Cu foil is fitted and the obtained amplitude reduction factor *S*_*0*_^*2*^ value (0.845) was set in the EXAFS analysis to determine the coordination numbers (CNs) in the Cu-O and Cu-Au scattering path in sample.

### Computational methods

DFT models were constructed based on the results of AC-HAADF-STEM and XRD, and the (101) crystal plane of TiO_2_ was selected to construct the computational models. All DFT calculations were performed by the Vienna Ab Initio Simulation Package (VASP). The Perdew-Burke-Ernzerhof (PBE) generalized gradient approximation (GGA) was utilized to address the exchange-correlation interactions. All slab models were applied with a 15 Å vacuum layer to prevent interactions between slabs. A 2 × 2 × 1 *k*-point was sampled in the Brillouin zone. A plane-wave basis expansion with a 400 eV energy cutoff was adopted to optimize the geometric structures, and the electronic forces were converged to 1 × 10^−5^ and 0.03 eV·Å^−1^, respectively. The Gibbs free energies were calculated at 298.15 K by means of the formula *G* = *E*_DFT_ − *TS* + *E*_ZPE_, where *E*_DFT_, *TS*, and *E*_ZPE_ represent the electronic energy of each step, entropy contribution, and zero-point energy, respectively.

### Supplementary information

Supplementary Information

Peer Review File

## Data Availability

All data that support the findings of this study are present in the paper and the Supplementary Information. Further information can be acquired from the corresponding authors. Source data are provided with this paper.
