# Supplementary material for: Well-defined diatomic catalysis for photosynthesis of C2H4 from CO2
Source: Nat Commun. 2024 Mar 18;15:2422. doi: 10.1038/s41467-024-46745-3 (PMC10948895; doi:10.1038/s41467-024-46745-3)
Supplement: Supplementary file 1 — Supplementary Information [file 41467_2024_46745_MOESM1_ESM.pdf]

Supplementary Information

Well-defined diatomic catalysis for photosynthesis of C_2H_4 from CO_2

Zhongkai Xie[1], Shengjie Xu[1], Longhua Li[1], Shanhe Gong[1], Xiaojie Wu[1], Dongbo Xu[1], Baodong Mao[1], Ting Zhou[1], Min Chen[1], Xiao Wang[2], Weidong Shi[1]✉, and Shuyan Song[2]✉

[1] School of Chemistry and Chemical Engineering, Jiangsu University, Zhenjiang 212013, China

[2] State Key Laboratory of Rare Earth Resource Utilization, Changchun Institute of Applied Chemistry, Chinese Academy of Sciences, Changchun 130022, China

E-mail address: swd1978@ujs.edu.cn; songsy@ciac.ac.cn

Contents:

Supplementary Figure 1-49

Supplementary Table 1-5

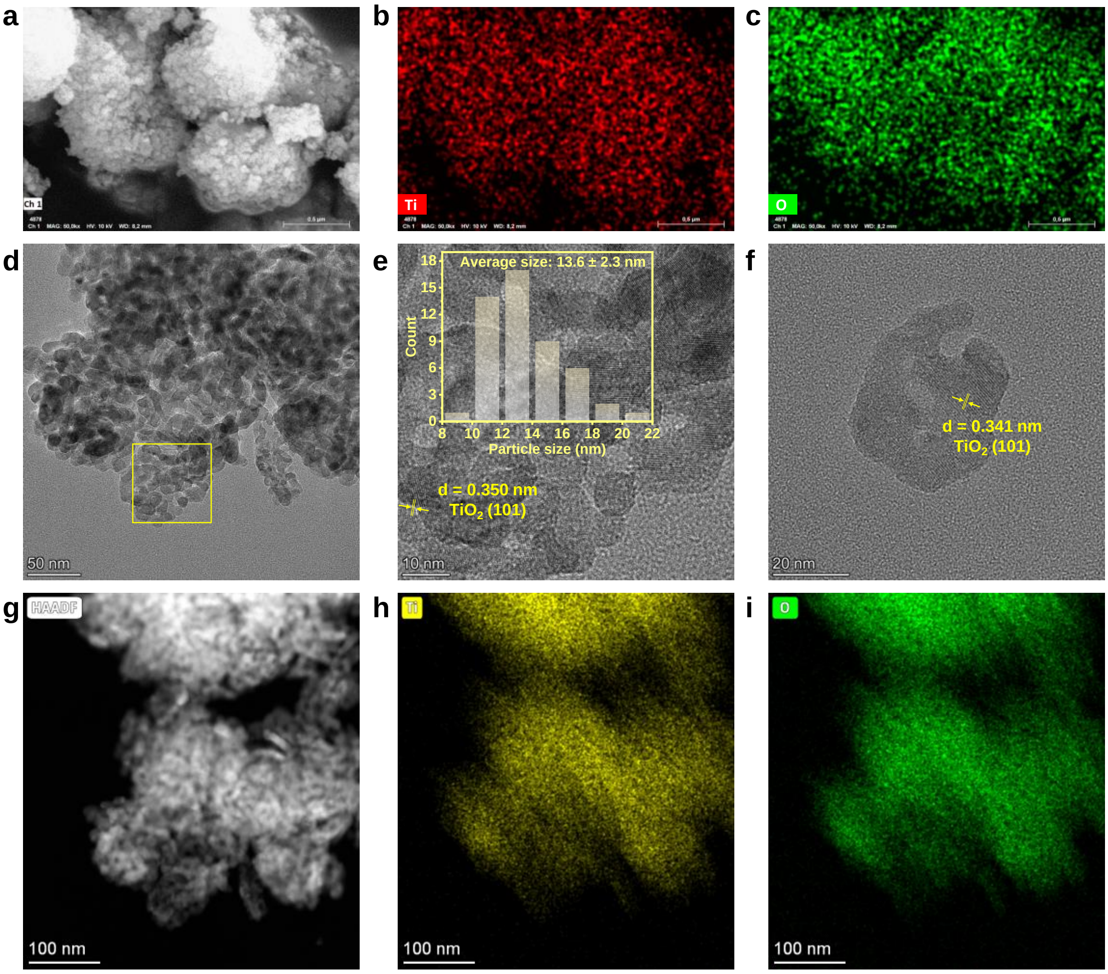

Supplementary Figure 1. Morphological structure characterization of TiO_2. **a**, SEM image of TiO_2. The corresponding EDS mapping images of Ti (**b**) and O (**c**) elements in (**a**). **d**, TEM image of TiO_2. **e**, HRTEM image and particle size distribution. **f**, HRTEM image of TiO_2. **g**, HAADF-STEM image of TiO_2. The corresponding EDS mapping images of Ti (**h**) and O (**i**) elements in (**g**).

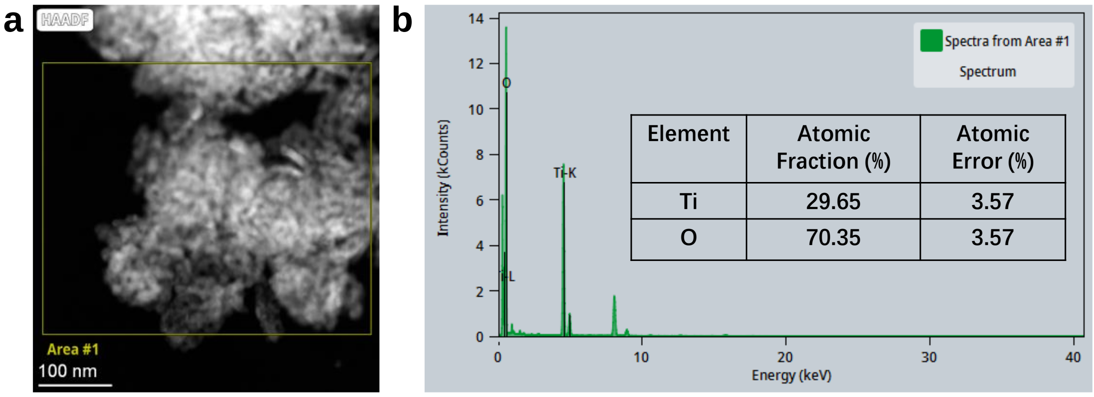

Supplementary Figure 2. Elemental distribution of TiO_2. **a**, HAADF-STEM image of TiO_2. **b**, EDS spectrum of the selected area in (**a**).

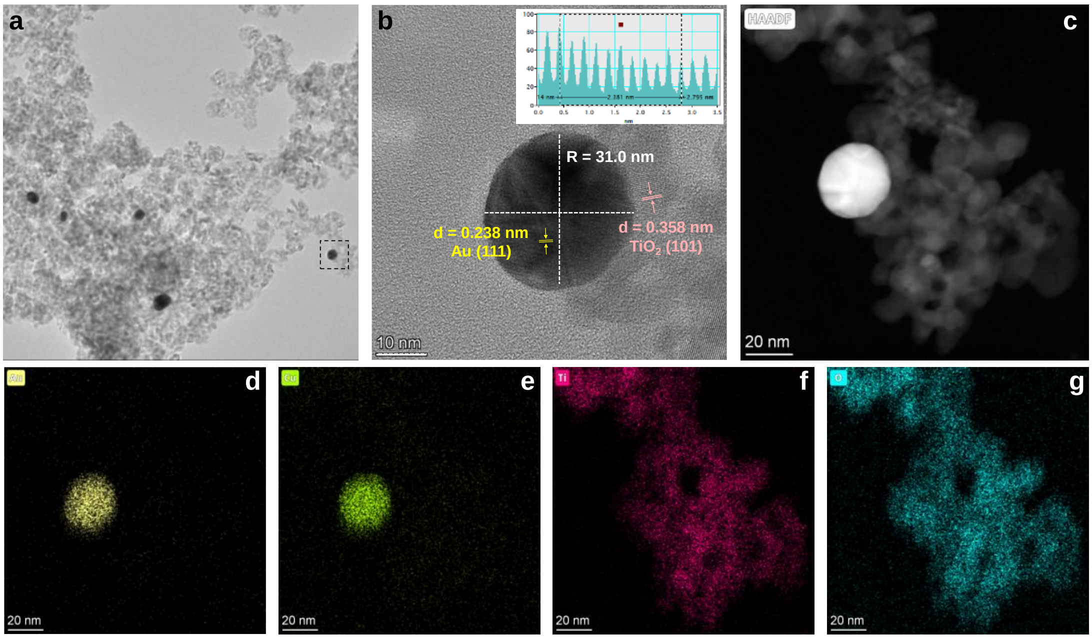

Supplementary Figure 3. Morphological structure characterization of Cu_5Au_1-TiO_2. **a**, TEM image of Cu_5Au_1-TiO_2. **b**, HRTEM image of the selected area in (**a**). **c**, HAADF-STEM image of Cu_5Au_1-TiO_2. The corresponding EDS mapping images of Au (**d**), Cu (**e**), Ti (**f**), and O (**g**) elements in (**c**).

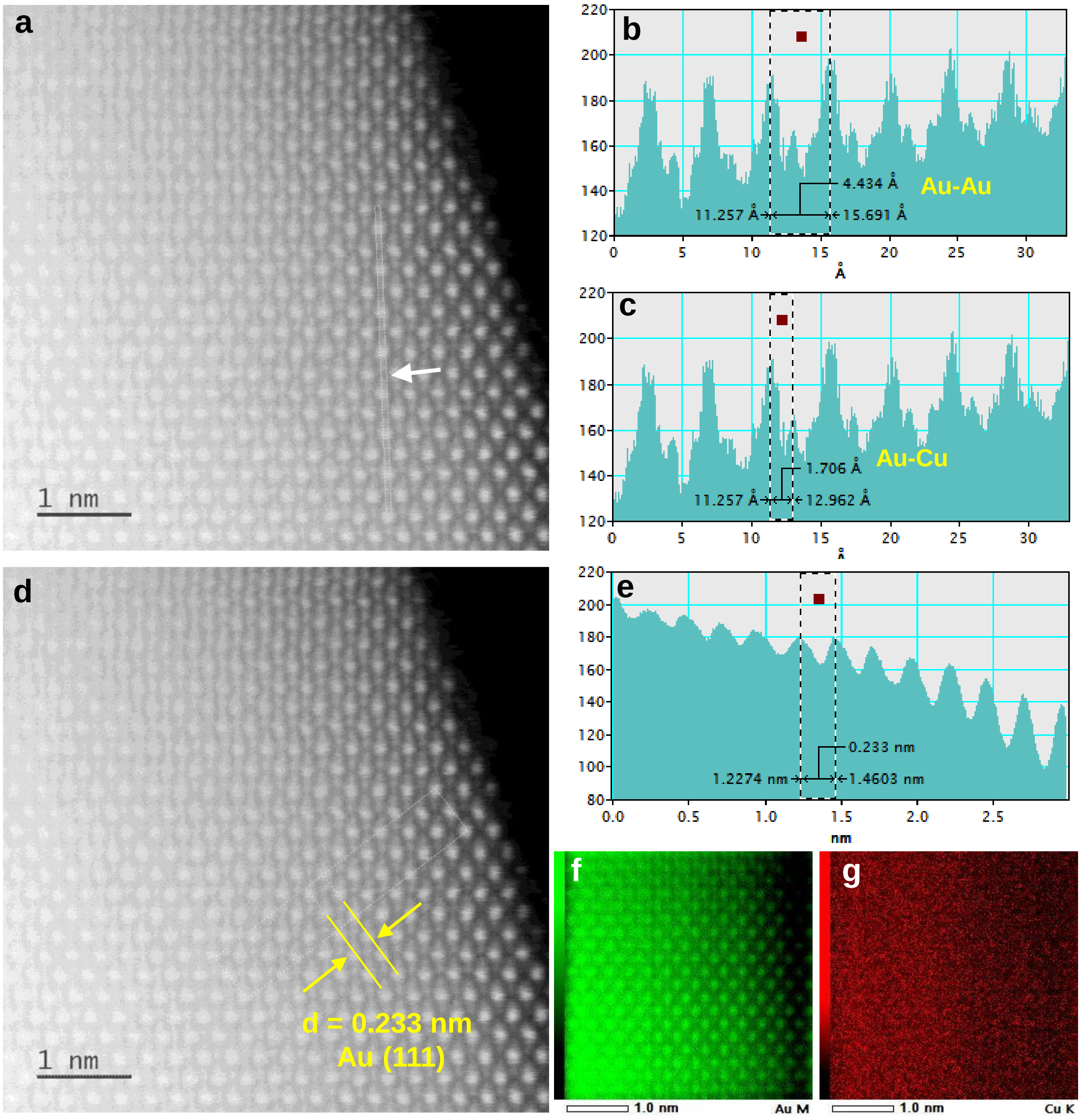

Supplementary Figure 4. Atomic-level configuration of Cu_5Au_1-TiO_2. Acquired AC-HAADF-STEM images intensity profile of Cu_5Au_1-TiO_2 (**a**) for the analysis of Au-Au (**b**) and Au-Cu (**c**) distance. Acquired lattice spacing of Cu_5Au_1 alloy (**d**) and corresponding intensity profile (**e**). The corresponding EDS mapping images of Au (**f**) and Cu (**g**) elements in (**d**).

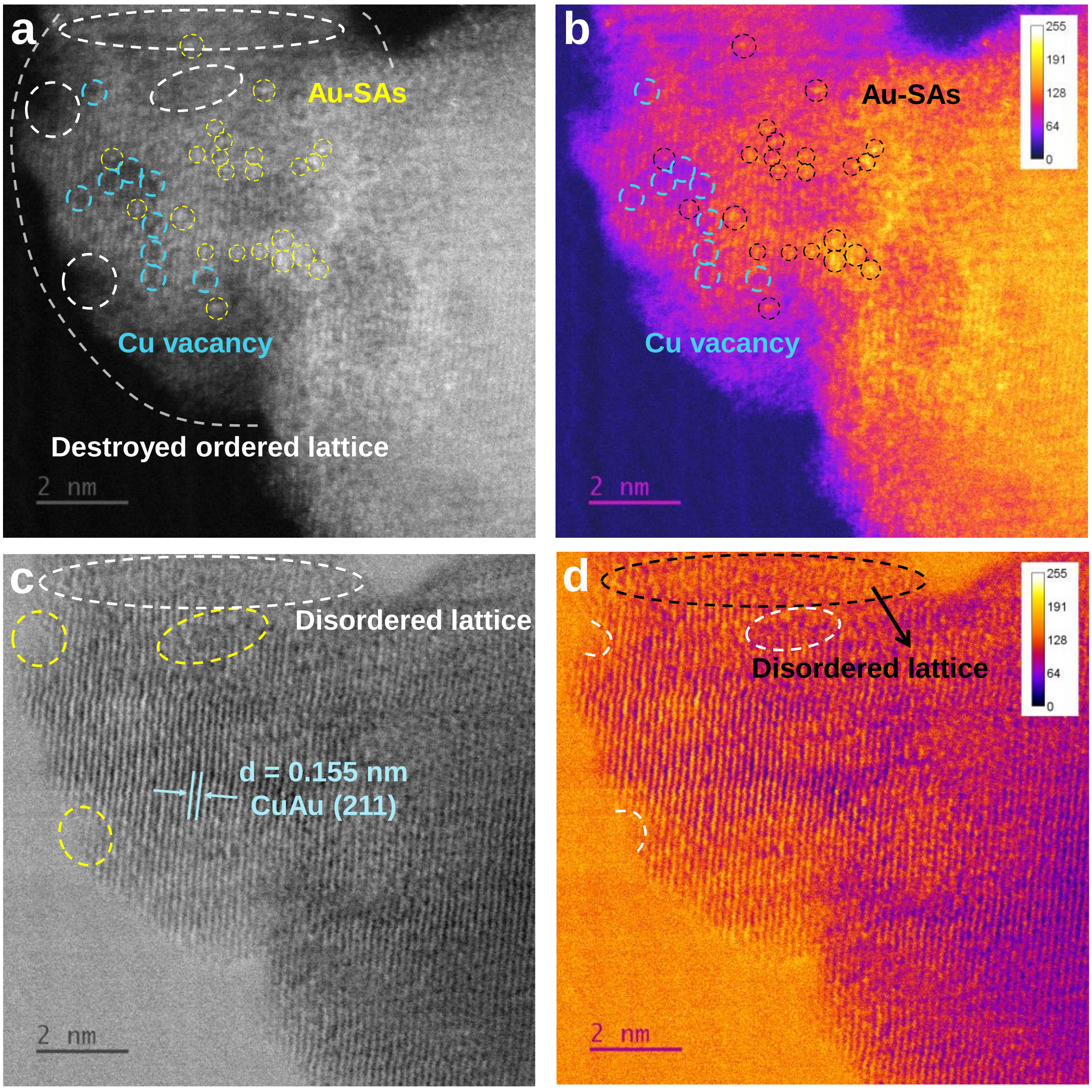

Supplementary Figure 5. Atomic-level configuration of E_1-Cu_5Au_1-TiO_2. AC-HAADF-STEM of E_1-Cu_5Au_1-TiO_2 (**a**) and the corresponding intensity-profile images of pseudo-color with calibration bar (**b**). HRTEM of E_1-Cu_5Au_1-TiO_2 (**c**) and the corresponding intensity-profile images of pseudo-color with calibration bar (**d**).

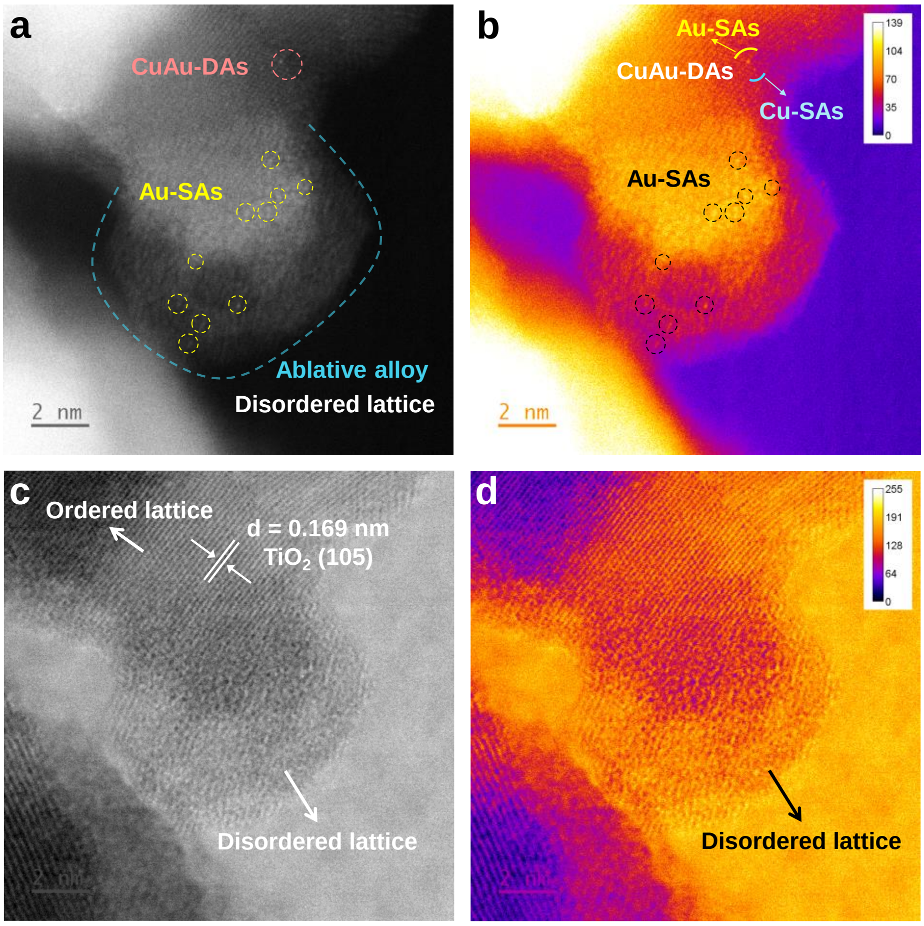

Supplementary Figure 6. Atomic-level configuration of E_3-Cu_5Au_1-TiO_2. AC-HAADF-STEM of E_3-Cu_5Au_1-TiO_2 (**a**) and the corresponding intensity-profile images of pseudo-color with calibration bar (**b**). HRTEM of E_3-Cu_5Au_1-TiO_2 (**c**) and the corresponding intensity-profile images of pseudo-color with calibration bar (**d**).

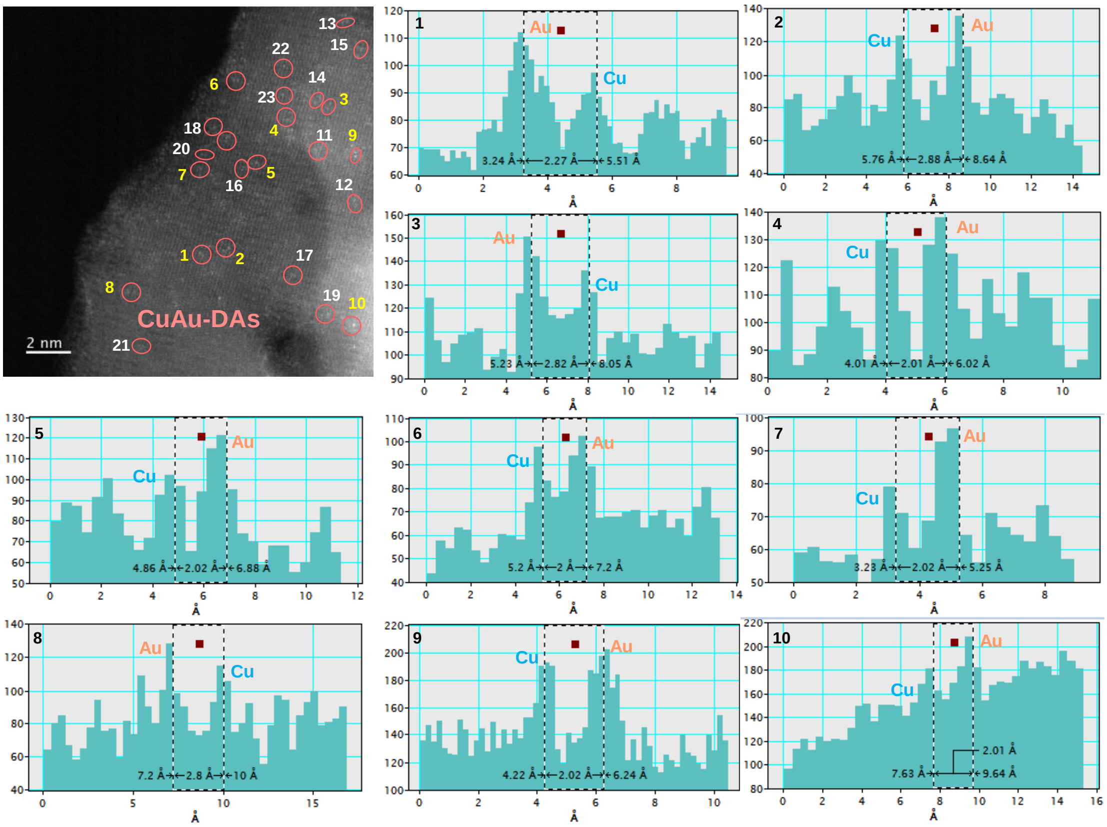

Supplementary Figure 7. Atomic-level configuration of E_7-Cu_5Au_1-TiO_2.

Acquired AC-HAADF-STEM images intensity profile of E_7-Cu_5Au_1-TiO_2.

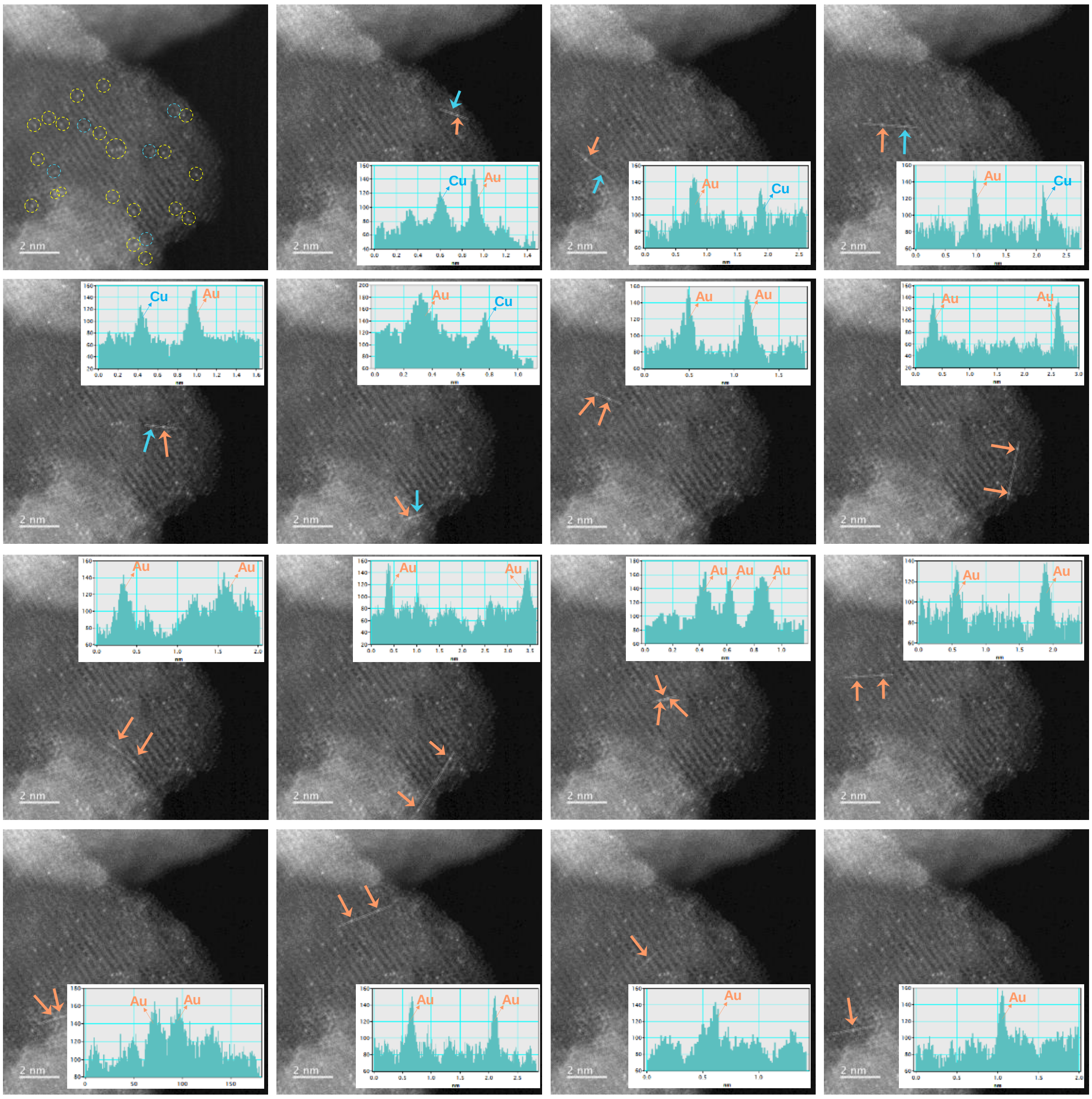

Supplementary Figure 8. Atomic-level configuration of E_{11}-Cu_5Au_1-TiO_2.

AC-HAADF-STEM images and intensity profile of E_{11}-Cu_5Au_1-TiO_2.

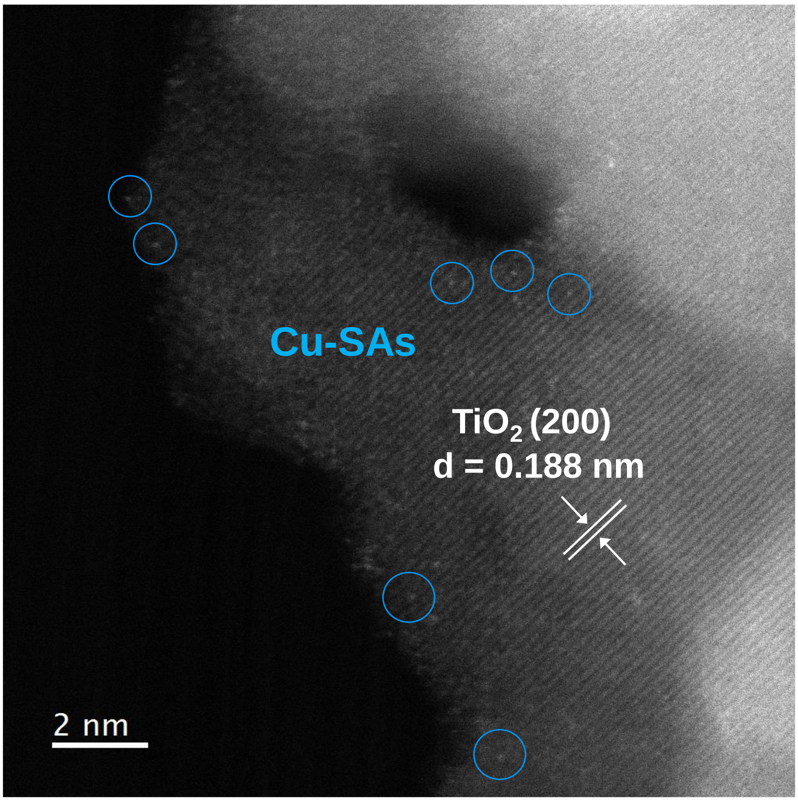

Supplementary Figure 9. Atomic-level configuration of E_7-Cu-TiO_2. AC-HAADF-STEM image of E_7-Cu-TiO_2.

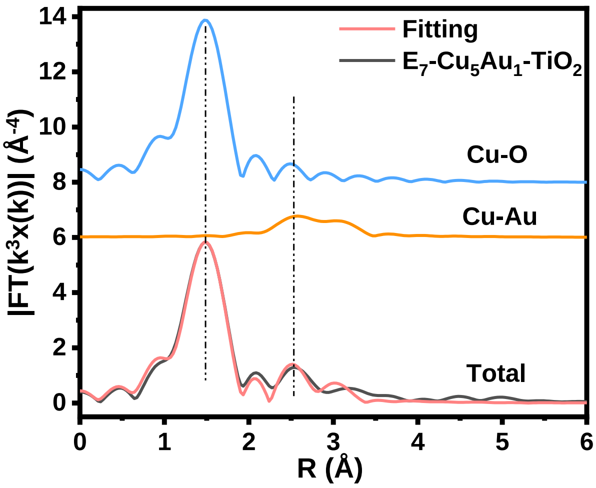

Supplementary Figure 10. The coordination environment of Cu atoms in E_7-Cu_5Au_1-TiO_2. Cu K-edge EXAFS fitting curves of E_7-Cu_5Au_1-TiO_2 in the R space.

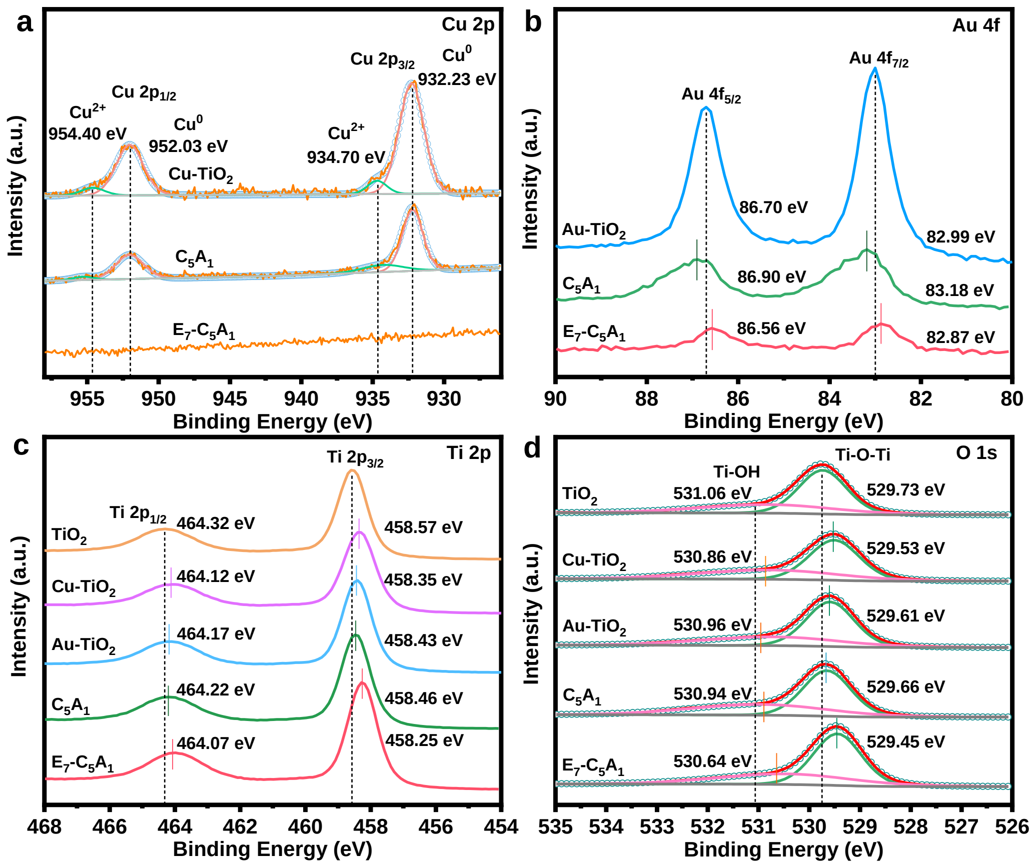

Supplementary Figure 11. XPS analysis of the as-prepared samples. Cu 2p (**a**), Au 4f (**b**), Ti 2p (**c**), and O 1s (**d**) XPS spectra of the as-prepared samples.

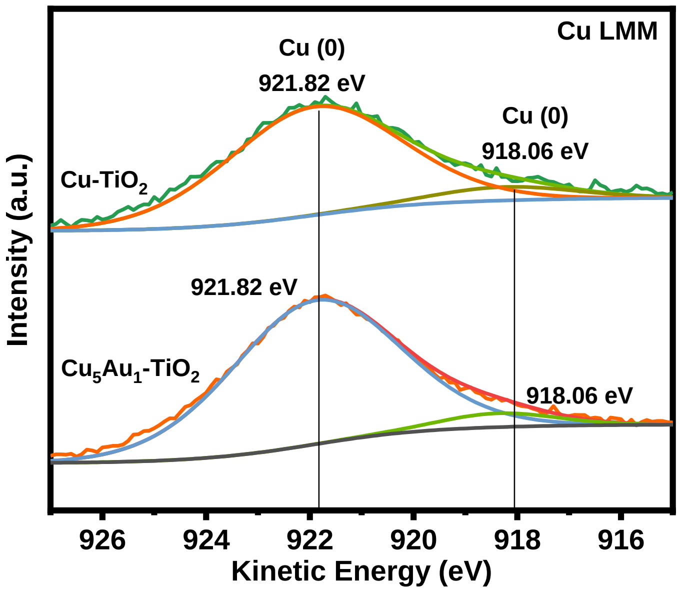

Supplementary Figure 12. Analysis of Cu valence. Auger spectrum (Cu LMM peak) of Cu-TiO_2 and Cu_5Au_1-TiO_2.

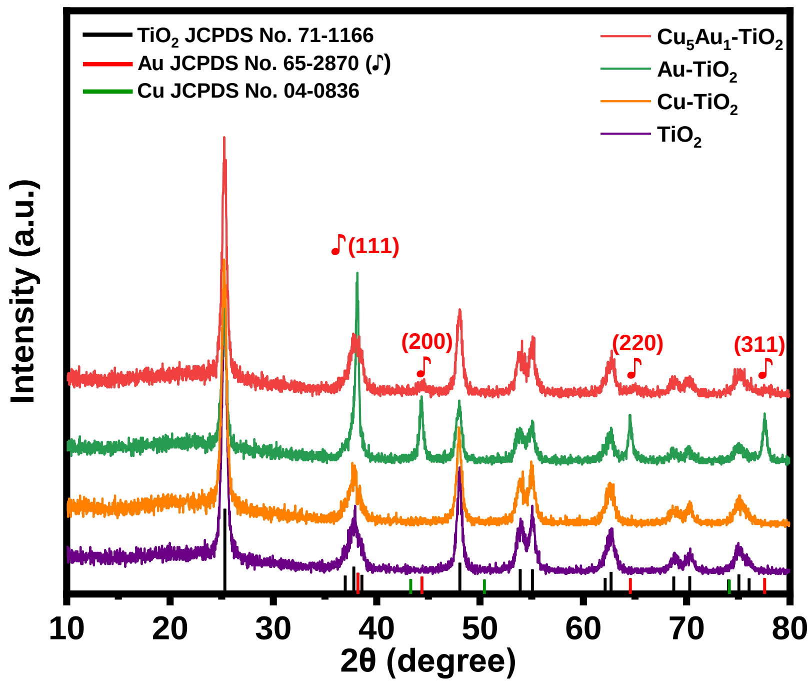

Supplementary Figure 13. Structural analysis by XRD. XRD patterns of TiO_2, Au-TiO_2, Cu-TiO_2, and Cu_5Au_1-TiO_2.

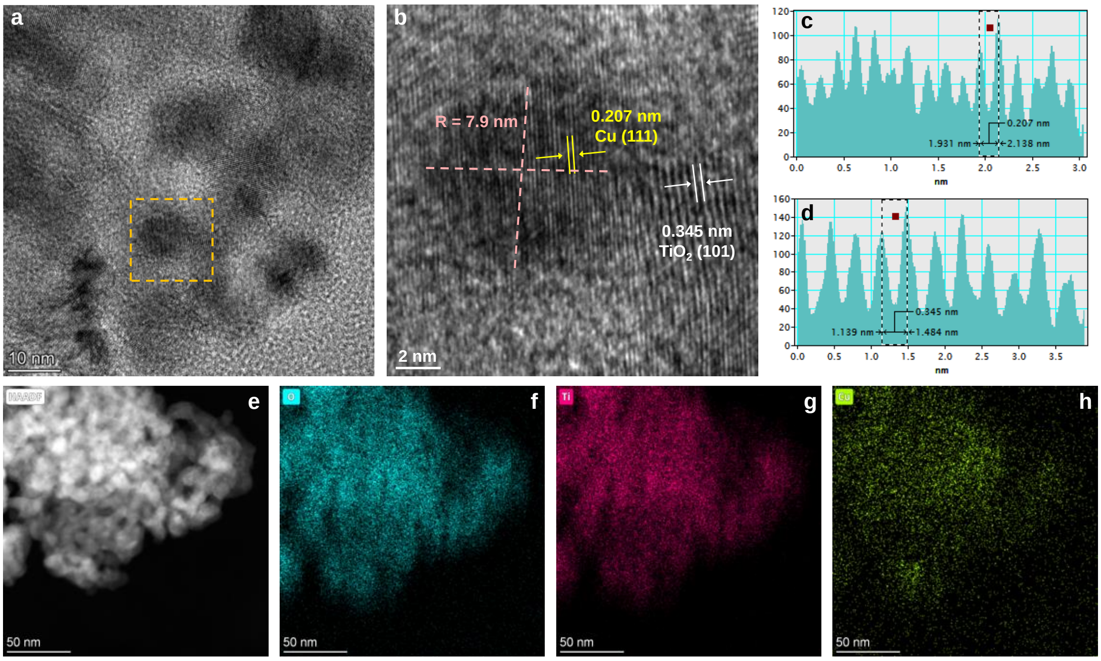

Supplementary Figure 14. Morphological structure characterization of Cu-TiO_2. **a**, HRTEM image of Cu-TiO_2. **b**, Magnifying HRTEM image of Cu-TiO_2 in (**a**). Acquired HRTEM images intensity profile of (**c**) and (**d**) in (**b**). **e**, HAADF-STEM image of Cu-TiO_2. The corresponding EDS mapping images of O (**f**), Ti (**g**), and Cu (**h**) in (**e**).

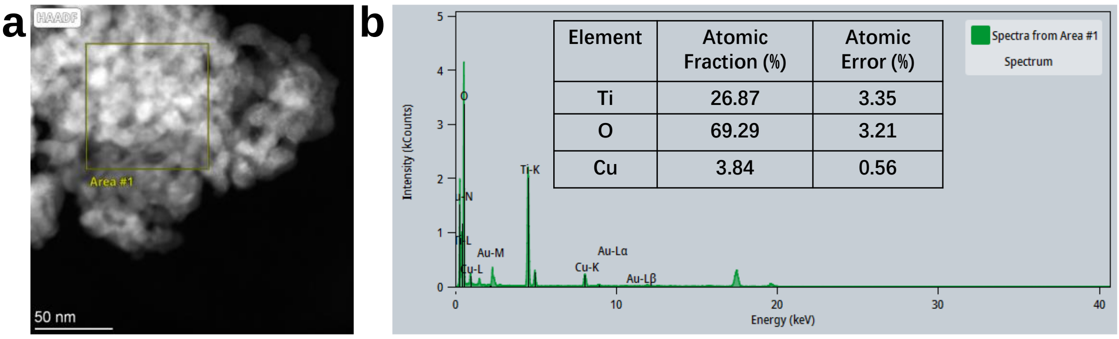

Supplementary Figure 15. Elemental distribution of Cu-TiO_2. **a**, HAADF-STEM image of Cu-TiO_2. **b**, EDS spectrum of the selected area in (**a**).

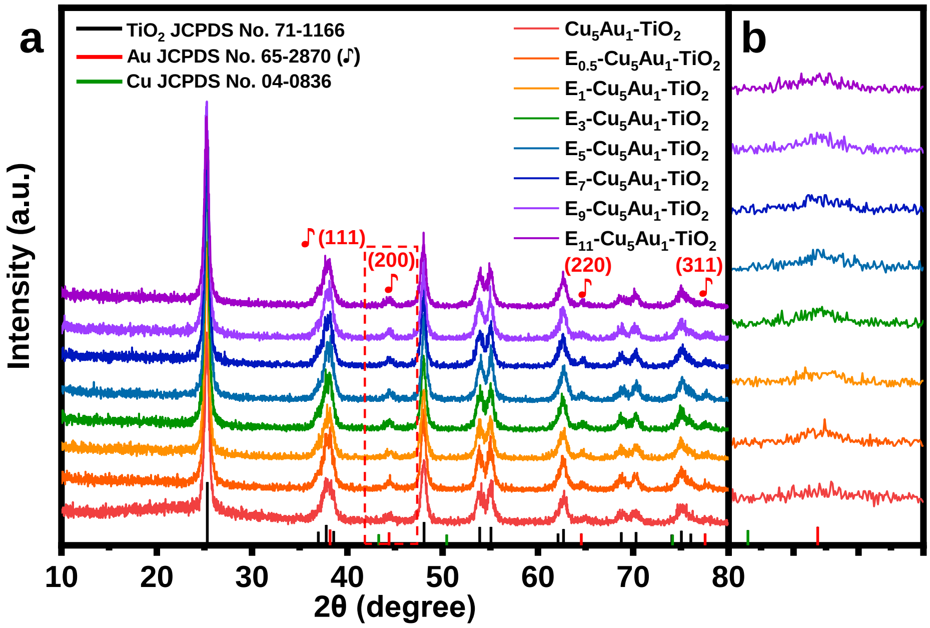

Supplementary Figure 16. Structural analysis by XRD. **a**, XRD patterns of Cu_5Au_1-TiO_2 with different vectored etching time. **b**, The corresponding magnification of the rectangle in (**a**).

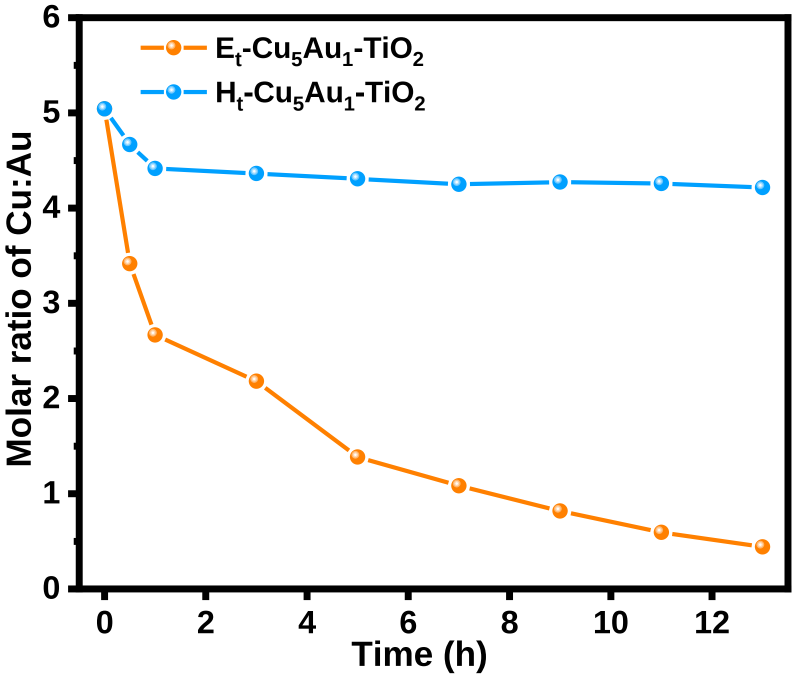

Supplementary Figure 17. Elemental distribution analysis by ICP-AES.

ICP-AES patterns of E_t-Cu_5Au_1-TiO_2 and H_t-Cu_5Au_1-TiO_2.

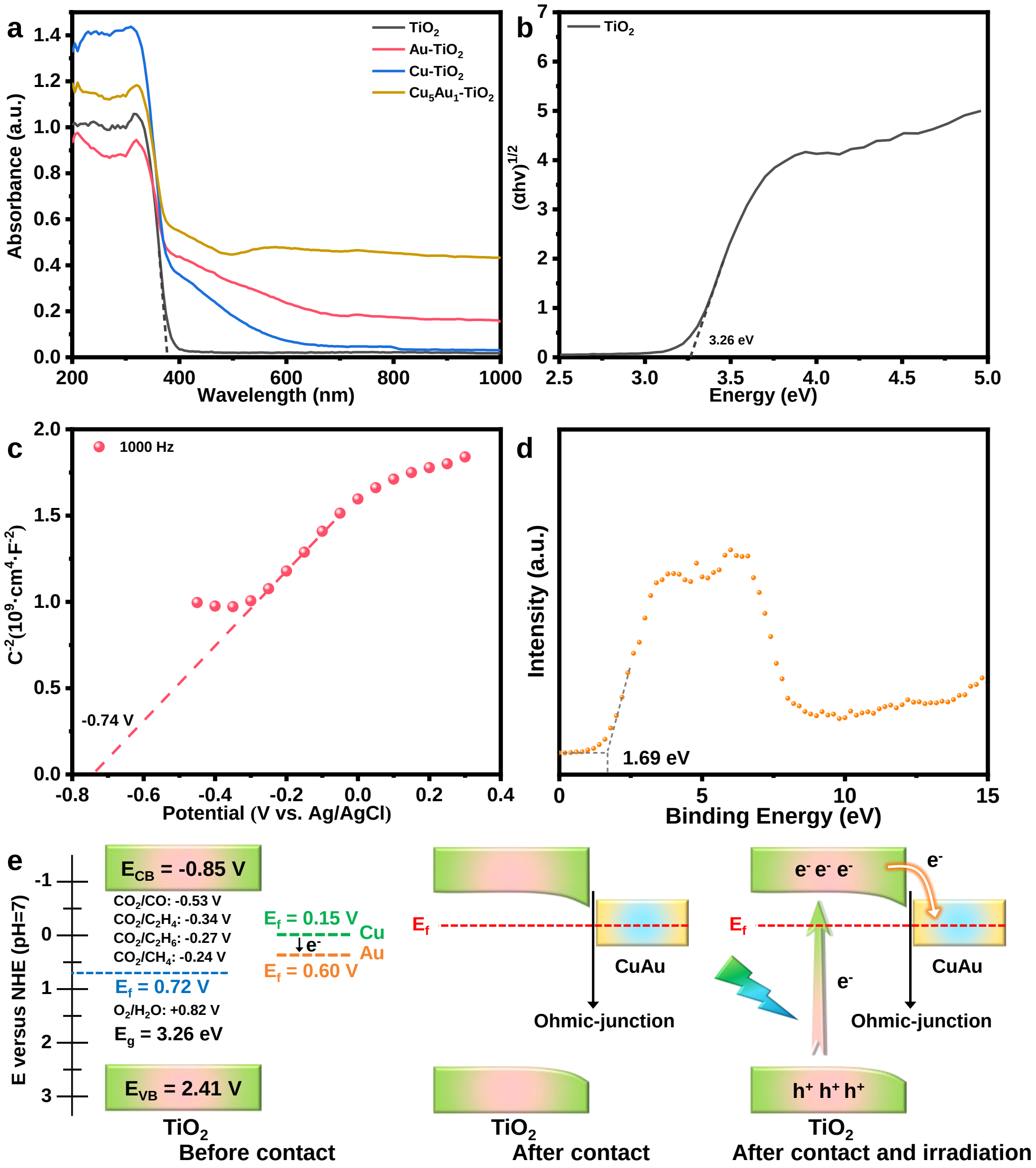

Supplementary Figure 18. Band structure and charge transfer dynamics analysis. **a**, UV-vis-NIR DRS spectra of as-prepared samples without etching. **b**, Plot of $(\alpha h\nu)^{1/2}$ versus $(h\nu)$ for the bandgap energy of TiO_2. **c**, Mott-Schottky plots of TiO_2. **d**, The survey XPS spectrum of valence band spectrum of TiO_2. **e**, Schematics illustrating the electronic band structure of TiO_2 before and after contact with CuAu.

Based on the data of UV-vis DRS spectra, the plot of $(\alpha h\nu)^{1/2}$ vs. hν was also fitted to further evaluate the bandgap energy of the as-prepared

samples, and the bandgap energy (E_g) of TiO_2 is 3.26 eV (Supplementary Fig. 18a,b). The Mott-Schottky (MS) show the flat band potential of TiO_2 was estimated to be -0.74 V vs. Ag/AgCl (Supplementary Fig. 18c), which was equal to -0.55 V vs. NHE ($E_{NHE} = E_{Ag/AgCl} + 0.197$ V). Based on the positive MS plot of TiO_2, TiO_2 was defined as n-type semiconductor, and the flat band potential is approximate 0.3 V below to the CB potential (E_{CB}) in n-type semiconductor[1,2], indicating the -0.85 V vs. NHE of E_{CB} potential[3,4]. Meanwhile, the VB potential (E_{VB}) of TiO_2 was further calculated to be 2.41 V. In Supplementary Fig. 18d, the VB-XPS spectra exhibited the 1.69 eV energy gap between the Fermi level (E_f) and VB, and the E_f could be calculated to be 0.72 V. It has been reported that the work functions of metal Cu and Au correspond to 4.65 and 5.10 eV[5], respectively, and the E_f of metal Cu and Au could be calculated to be 0.15 and 0.60 V by the formula of E (vs. NHE, pH = 0) = -4.5 eV – E (vacuum level)[6], indicating that the electron could further transfer from Cu to the lower E_f of Au (Supplementary Fig. 18e). Based on the difference of E_f between Cu and Au, the more efficient photogenerated charge carrier separation could be realized on the CuAu modified TiO_2 compared to Cu modified TiO_2. Due to the larger E_f value of TiO_2 compared to CuAu metal, the electrons would transfer from CuAu to TiO_2 until their Fermi levels are aligned to the equilibrium state, and the ohmic-junction could be further fabricated to provide the highspeed photogenerated charge carrier transfer pathways[7,8].

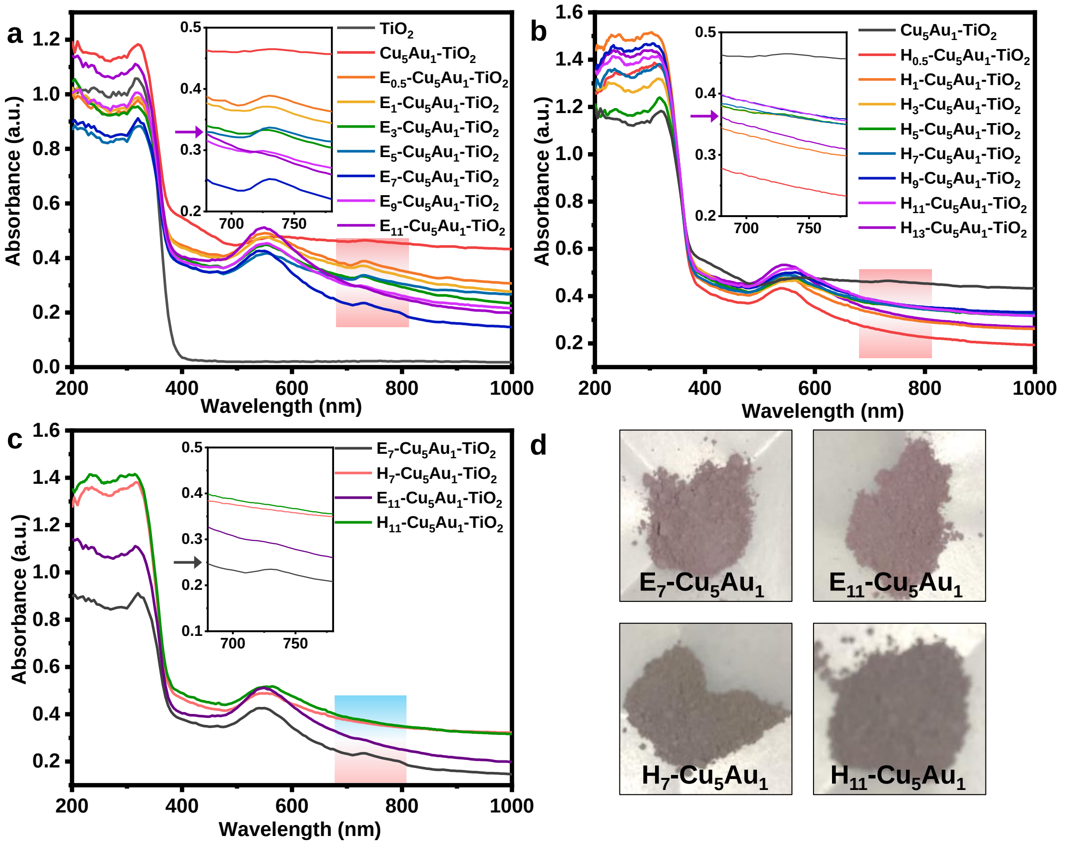

Supplementary Figure 19. Structural and photo absorption analysis by UV-vis-NIR DRS spectra. UV-vis-NIR DRS spectra of Cu_5Au_1-TiO_2 with different vectored etching (**a**) and pure HCl etching (**b**) time. **c**, UV-vis-NIR DRS comparation of Cu_5Au_1-based samples under different etching methods. **d**, Real-time photographs of Cu_5Au_1-based samples.

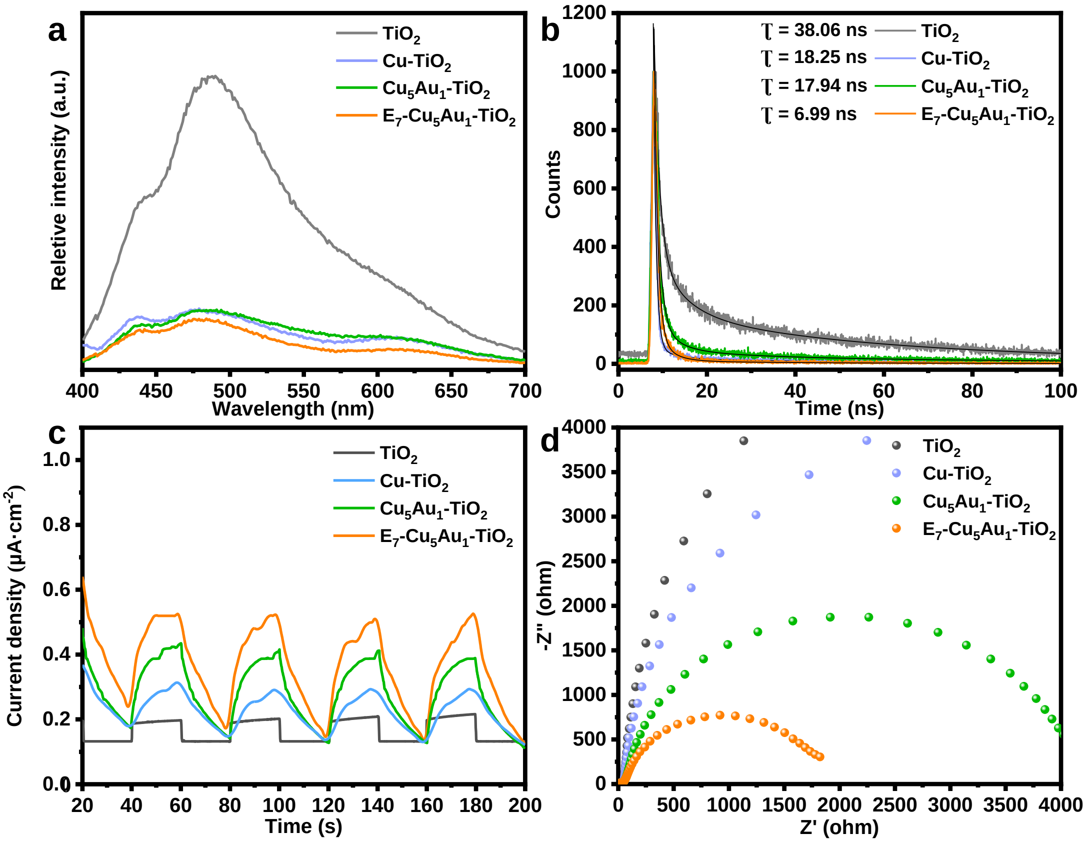

Supplementary Figure 20. Dynamics analysis of photogenerated charge carrier. **a**, Steady-state photoluminescence spectra of the as-prepared samples. **b**, Time-resolved fluorescence decay spectra of the as-prepared samples. **c**, Transient photocurrent response of as-prepared catalysts. **d**, EIS of as-prepared catalysts.

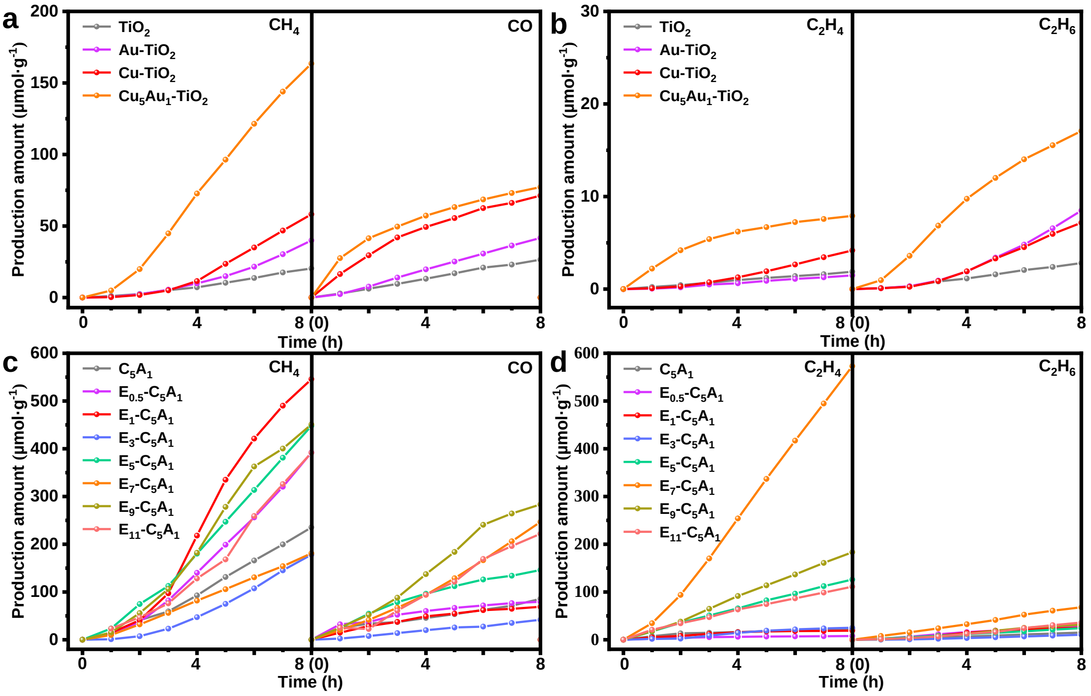

Supplementary Figure 21. Photocatalytic performance in detail. Time-dependent CO, CH_4, C_2H_4, and C_2H_6 evolution over as-prepared samples.

As shown in Supplementary Figure 21a, TiO_2, Au-TiO_2, Cu-TiO_2, and Cu_5Au_1-TiO_2 all display the linearly increased CH_4 production, while the Cu-TiO_2 and Cu_5Au_1-TiO_2 perform the non-linearly increased CO production, which implies that large contents of Cu could promote the poor stability for CO generation. In Supplementary Figure 21b, Cu_5Au_1 alloy modified TiO_2 displays the highest C_2H_4 and C_2H_6 production, which could be ascribed to the synergistic effect between Cu and Au components for the enhancement of C-C coupling. After the vectored etching of Cu, the CH_4 and CO production of E_t-Cu_5Au_1-TiO_2 increase hugely compared to that of Cu_5Au_1-TiO_2 (Supplementary Figure 21c), and the stability of CO production is significantly optimized, which indicates that the low-coordination Au and Cu atoms could promote the rapid CO desorption and

suppress the deactivation of photocatalysts. Moreover, an apparent increased C_2H_4 production is also observed in Supplementary Figure 21d, and both of the C_2H_4 and C_2H_6 production increase linearly, which further suggests that such low-coordination Cu and Au could benefit for the rapid CO generation, CO coupling, protonation, and accessible C_2 products desorption.

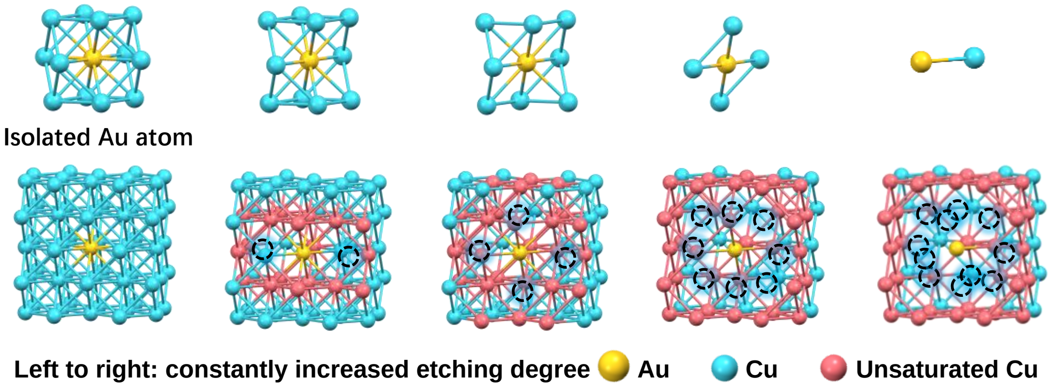

Supplementary Figure 22. Relationship between photocatalytic performance and photocatalysts structure. The possible schematic illustration in the evolution of Cu and Au sites under the constantly increased vectored etching treatment time.

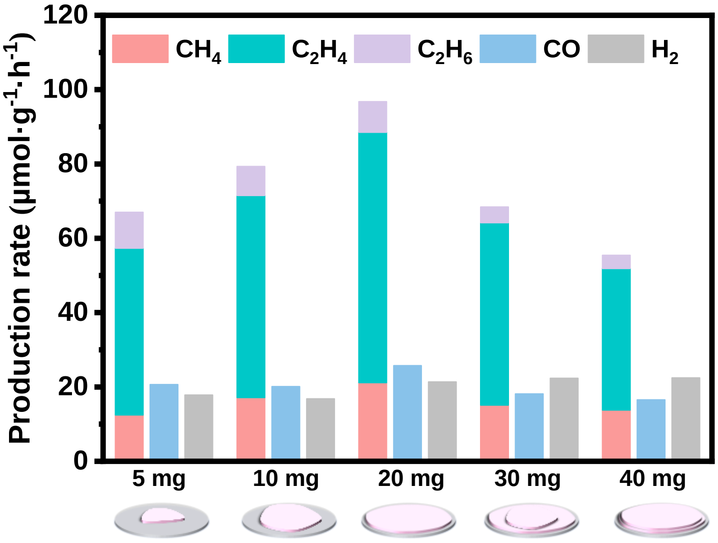

Supplementary Figure 23. Relationship between photocatalytic performance and photocatalysts contents. Dependence of the photocatalytic CO_2 reduction rate over different amounts of E_7-Cu_5Au_1-TiO_2.

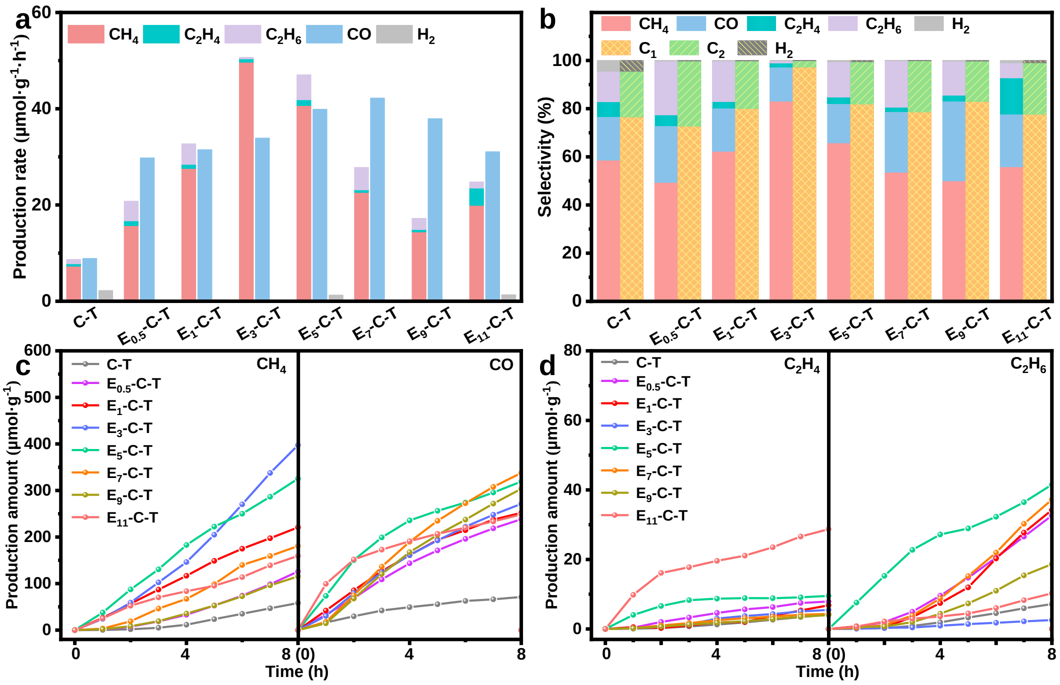

Supplementary Figure 24. Photocatalytic performance of E_t-Cu-TiO_2 in detail. **a**, CH_4, C_2H_4, C_2H_6, CO, and H_2 production rates of E_t-Cu-TiO_2. **b**, Electron-based selectivity of photocatalytic CO_2 conversion over E_t-Cu-TiO_2. **c**, Time-dependent CO and CH_4 evolution over E_t-Cu-TiO_2. **d**, C_2H_4, and C_2H_6 evolution over E_t-Cu-TiO_2.

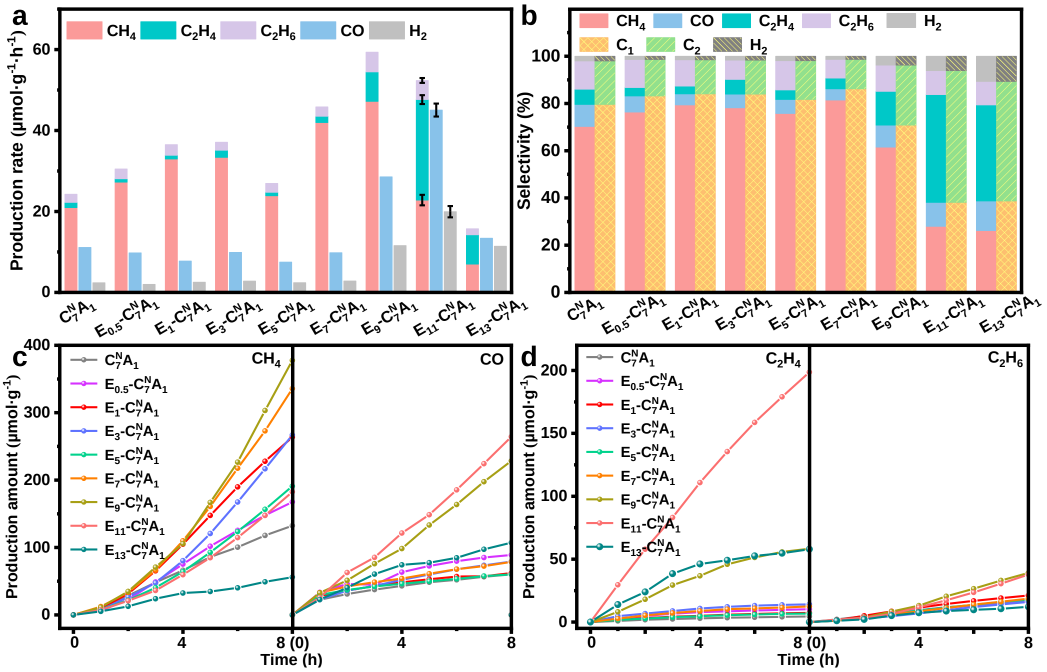

Supplementary Figure 25. Photocatalytic performance of E_t-Cu_7^N Au_1-TiO_2 in detail. **a**, CH_4, C_2H_4, C_2H_6, CO, and H_2 production rates of E_t-Cu_7^N Au_1-TiO_2. **b**, Electron-based selectivity of photocatalytic CO_2 conversion over E_t-Cu_7^N Au_1-TiO_2. **c**, Time-dependent CO and CH_4 evolution over E_t-Cu_7^N Au_1-TiO_2. **d**, C_2H_4, and C_2H_6 evolution over E_t-Cu_7^N Au_1-TiO_2.

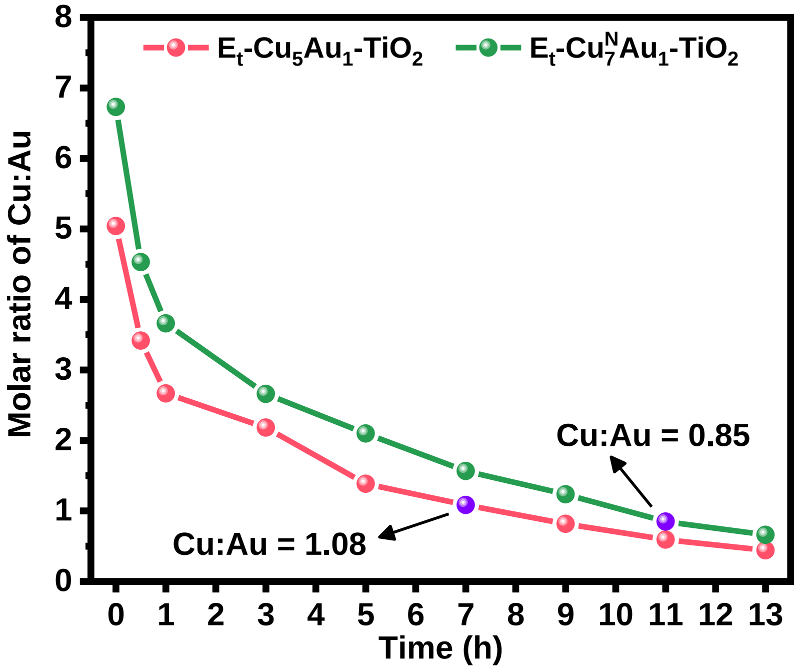

Supplementary Figure 26. Elemental distribution analysis by ICP-AES. ICP-AES patterns of E_t-$Cu_7^N Au_1$-TiO_2 and E_t-Cu_5Au_1-TiO_2.

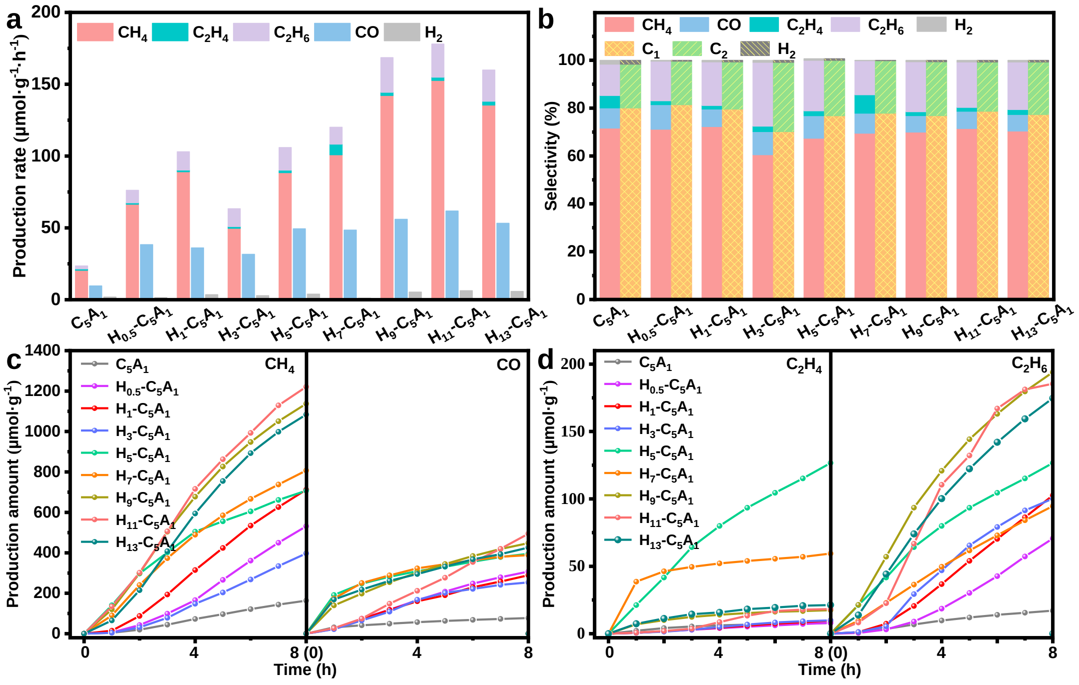

Supplementary Figure 27. Photocatalytic performance of H_t-Cu_5Au_1-TiO_2 in detail. **a**, CH_4, C_2H_4, C_2H_6, CO, and H_2 production rates of H_t-Cu_5Au_1-TiO_2. **b**, Electron-based selectivity of photocatalytic CO_2 conversion over H_t-Cu_5Au_1-TiO_2. **c**, Time-dependent CO and CH_4 evolution over H_t-Cu_5Au_1-TiO_2. **d**, C_2H_4, and C_2H_6 evolution over H_t-Cu_5Au_1-TiO_2.

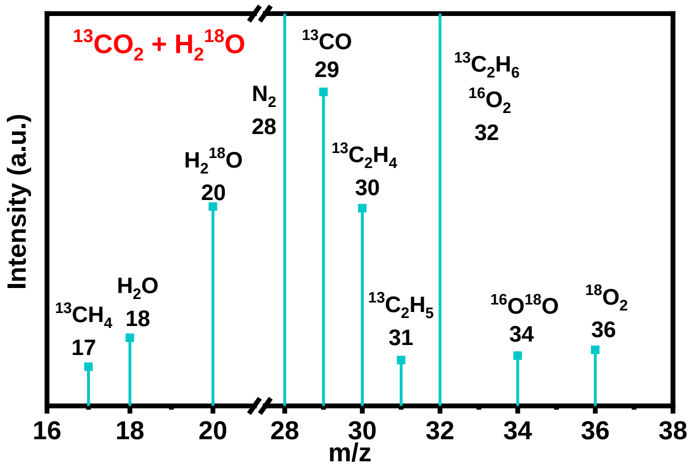

Supplementary Figure 28. Analysis of photoconversion CO_2 and H_2O mechanism. GC-MS spectra of CO, CH_4, C_2H_4, and C_2H_6 from the photocatalytic reduction of the $^{13}CO_2$ and $H_2{}^{18}O$ isotope labeling experiments on E_7-Cu_5Au_1-TiO_2.

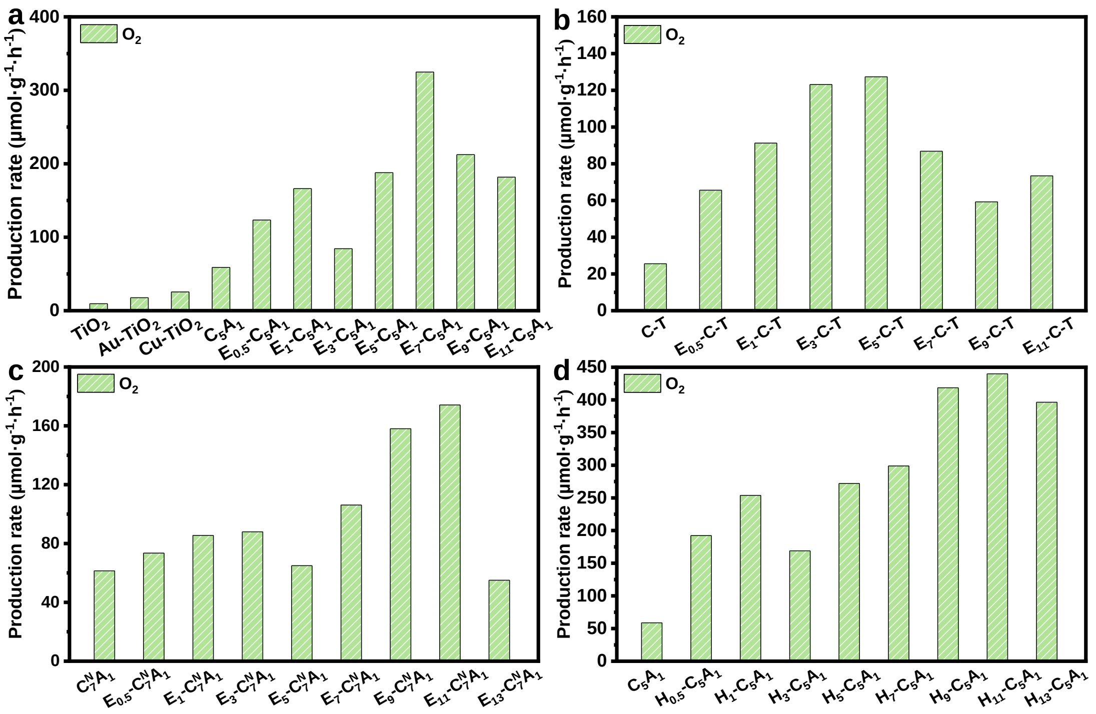

Supplementary Figure 29. Analysis of H_2O oxidation mechanism. O_2 production rates of different TiO_2-based samples.

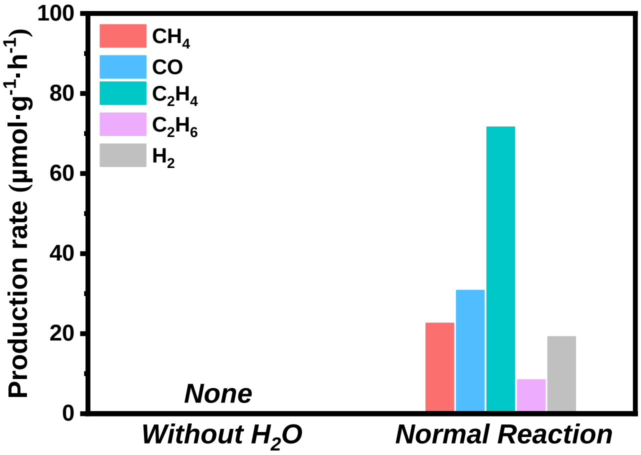

Supplementary Figure 30. Analysis of photoconversion CO_2 and H_2O mechanism. Comparison of CO_2 photoreduction with or without H_2O addition over E_7-Cu_5Au_1-TiO_2.

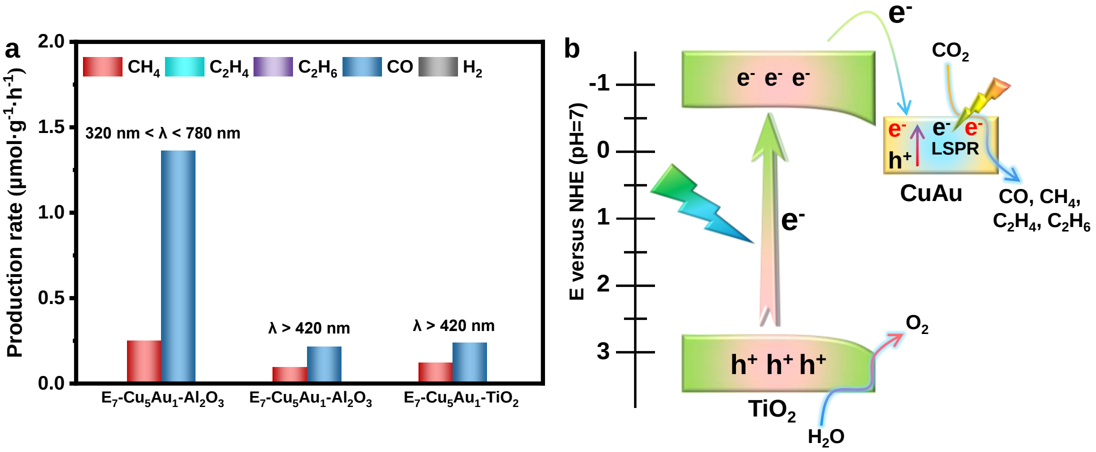

Supplementary Figure 31. Relationship between photocatalytic performance and irradiation source. **a**, CH_4, C_2H_4, C_2H_6, CO, and H_2 production rates of E$_7$-Cu$_5$Au$_1$-Al$_2$O$_3$ and E$_7$-Cu$_5$Au$_1$-TiO$_2$ under different irradiated wavelengths. **b**, Schematic illustration of E$_7$-Cu$_5$Au$_1$-TiO$_2$ for photoreduction CO_2.

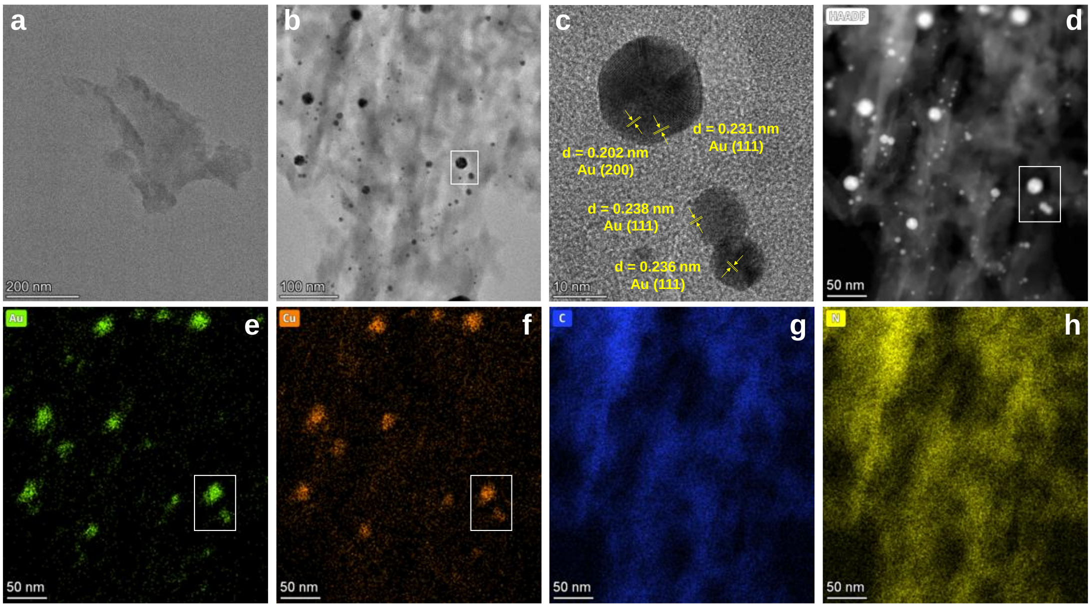

Supplementary Figure 32. Morphological structure characterization of TiO_2 and Cu_5Au_1-C_3N_4. **a**, TEM image of C_3N_4. **b**, TEM image of Cu_5Au_1-C_3N_4. **c**, HRTEM image of Cu_5Au_1-C_3N_4 that magnified in (**b**). **d**, HAADF-STEM image of Cu_5Au_1-C_3N_4. **e-h**, The corresponding EDS mapping images of Cu_5Au_1-C_3N_4 in (**d**).

The CuAu alloy nanoparticle was uniformly dispersed on the surface of C_3N_4 nanosheet (Supplementary Fig. 32a,b), and the lattice spacing of 0.202, 0.231, 0.238, and 0.236 are ascribed to (200), (111), (111), and (111) plane of Au (Supplementary Fig. 32c). However, HAADF-STEM (Supplementary Fig. 32d) and the corresponding elemental mapping (Supplementary Fig. 32e-h) verify the superimposed Cu and Au elements distribution, indicating the existence of CuAu alloy.

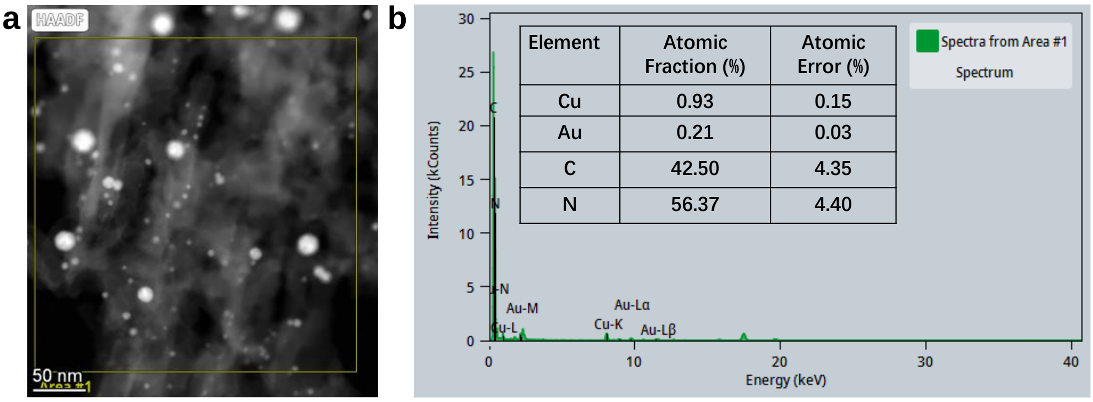

Supplementary Figure 33. Elemental distribution of Cu_5Au_1-C_3N_4. **a**, HAADF-STEM image of Cu_5Au_1-C_3N_4. **b**, EDS spectrum of the selected area in (**a**).

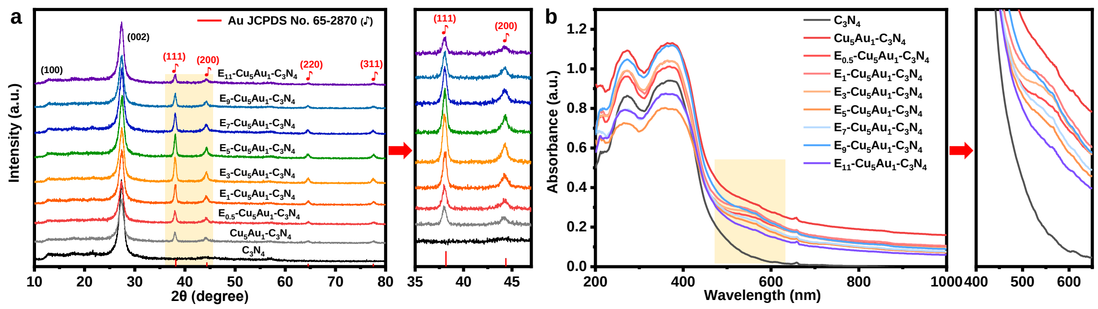

Supplementary Figure 34. Structural analysis by XRD and UV-vis-NIR DRS. **a**, XRD patterns of Cu_5Au_1-C_3N_4 with different vectored etching time. **b**, UV-vis-NIR DRS spectra of Cu_5Au_1-C_3N_4 with different vectored etching time.

Despite the EDS analysis (Supplementary Fig. 34) displays the 4.42 of Cu/Au atomic ratio, there is still no Cu lattice exhibited due to the nondominated Miller indexes of Cu phase (Supplementary Fig. 34a). The characteristic peaks at ca. 12.8 (100) and 27.5° (002) are attributed to the similar parallel diffraction modes and in-plane structure with pristine C_3N_4[9,10], indicating the successful fabrication of C_3N_4. Notably, the intensity of Au XRD characteristic peaks and Au LSPR response first increase and then decrease (Supplementary Fig. 34a,b), which implies that more Au sites were exposed due to the vectored etching of Cu elements, while the Au lattice was final to disappear and distribute as Au single atoms based on the constant decomposition of Cu lattice.

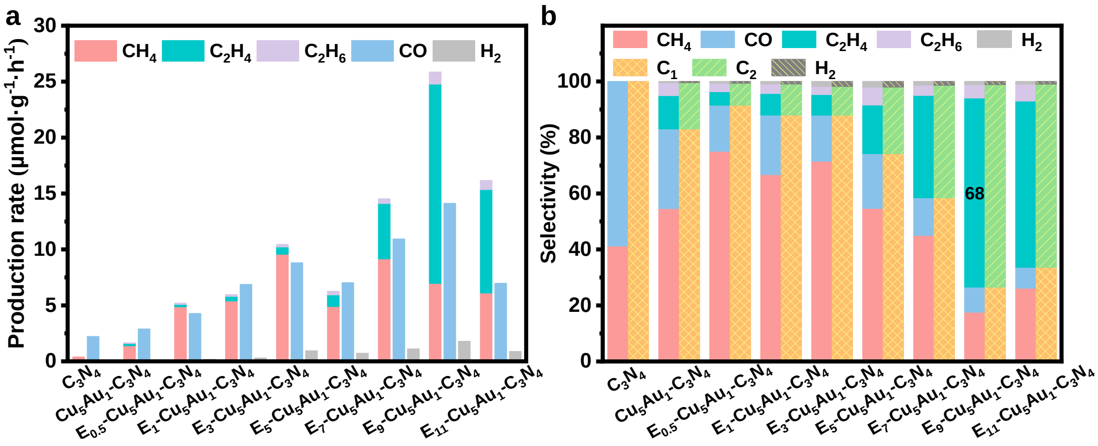

Supplementary Figure 35. Photocatalytic performance analysis of E_t-Cu_5Au_1-C_3N_4. **a**, CH_4, C_2H_4, C_2H_6, CO, and H_2 production rates of E_t-Cu_5Au_1-C_3N_4. **b**, Electron-based selectivity of photocatalytic CO_2 conversion over E_t-Cu_5Au_1-C_3N_4.

To further verify the universality of such optimized strategy for CO_2 photoreduction into C_2H_4, the CO_2 photoreduction tests were also conducted on C_3N_4-based samples under 320-780 nm irradiation (Supplementary Fig. 35a). After 9 h vectored etching of Cu in Cu_5Au_1-C_3N_4, the E_9-Cu_5Au_1-C_3N_4 exhibits the highest C_2H_4 production at a rate of 17.8 $\mu mol \cdot g^{-1}h^{-1}$, and the selectivity of C_2H_4 reaches up to 68% (Supplementary Fig. 35b), which also suggests that the low-coordination adjacent Cu and Au atoms could benefit for the C-C coupling and C_2H_4 production. Consequently, both the vectored etching Cu_5Au_1 modified C_3N_4 and TiO_2 exhibit the high C_2H_4 selectivity, which sufficiently prove the universality of such CuAu heteronuclear diatomics modification for the optimization of C-C coupling reaction.

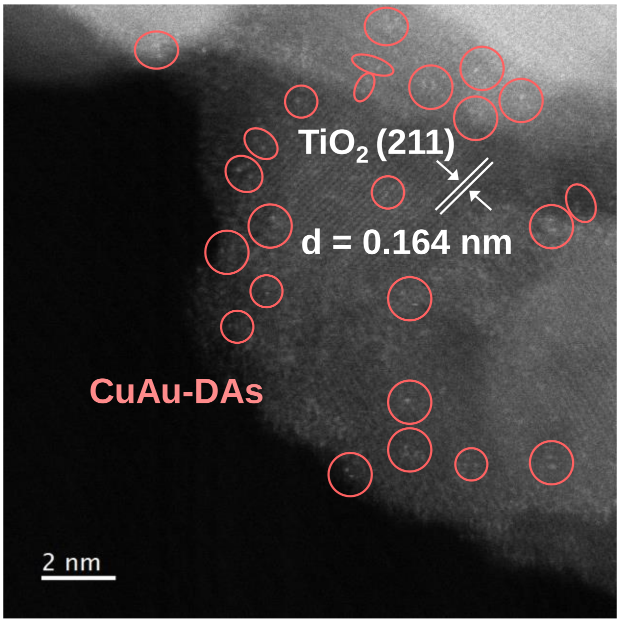

Supplementary Figure 36. Atomic-level configuration of E_7-Cu_5Au_1-TiO_2 after photocatalytic stability test. AC-HAADF-STEM image of E_7-Cu_5Au_1-TiO_2 after 72 h photocatalytic CO_2 reduction test.

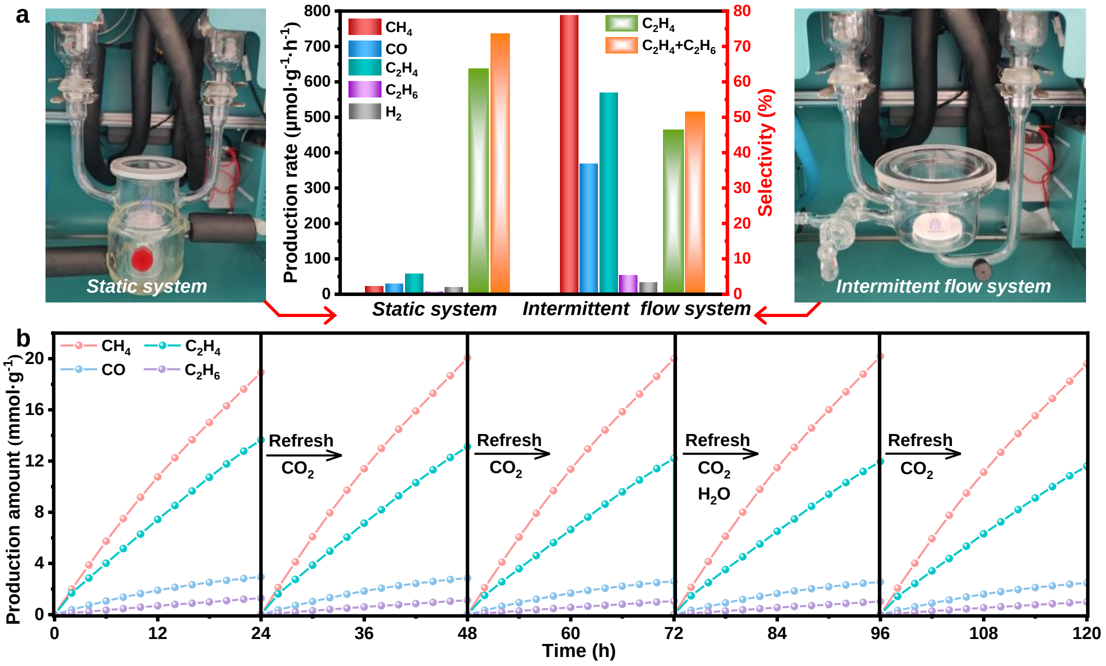

Supplementary Figure 37. Comparation of photocatalytic performance between different reaction systems. **a**, CH_4, C_2H_4, C_2H_6, CO, and H_2 production rates of E_7-Cu_5Au_1-TiO_2 under different reaction system over 24 h. **b**, Long-time photocatalytic stability test of E_7-Cu_5Au_1-TiO_2 in the intermittent flow system.

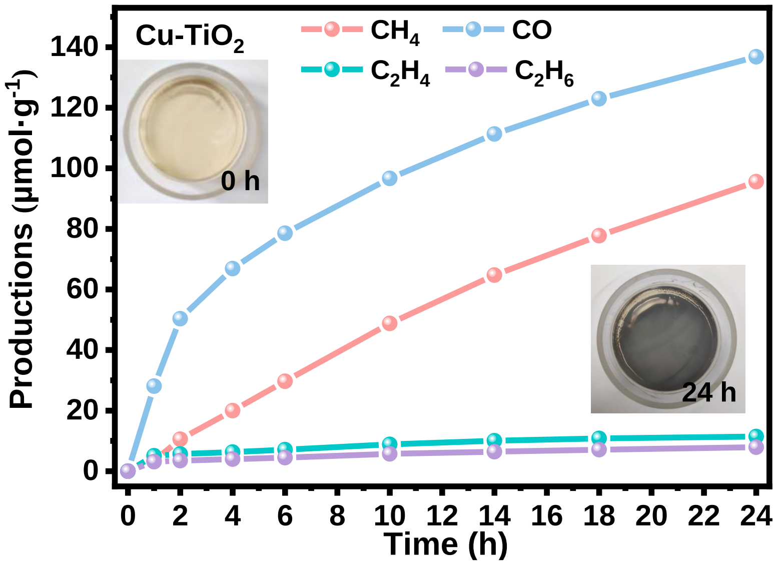

Supplementary Figure 38. Photocatalytic stability test of Cu-TiO_2. Long-time photocatalytic stability test of Cu-TiO_2.

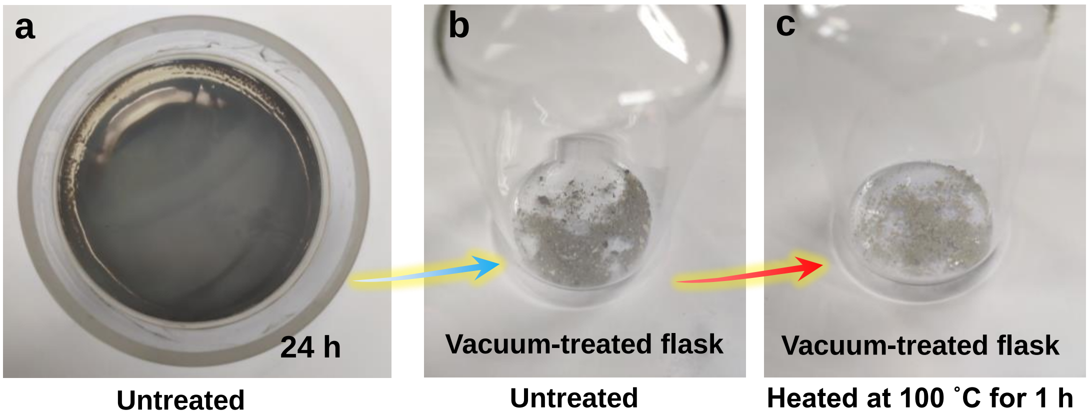

Supplementary Figure 39. Real-time photographs of photocatalysts before and after photocatalytic stability tests. **a,b**, Real-time photographs of Cu-TiO_2 collected from the quartz glass after the 24 h photocatalytic CO_2 reduction. **c**, Real-time photographs of Cu-TiO_2 heated for 1 h at 100 °C in muffle furnace.

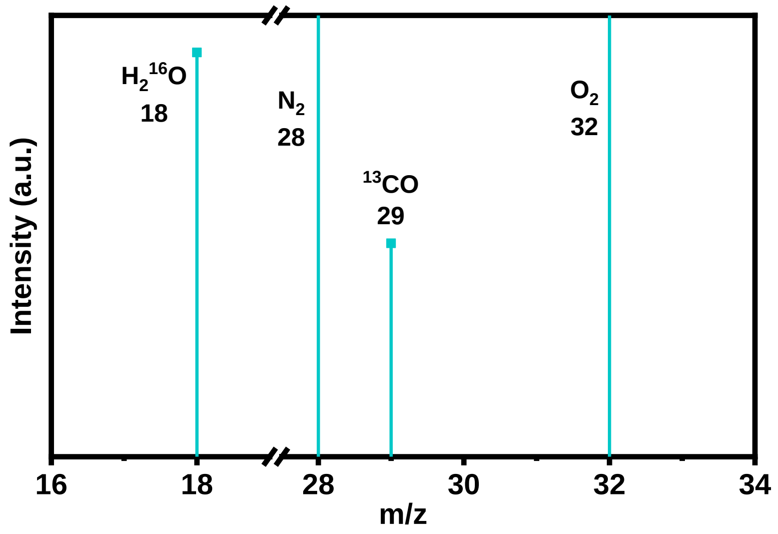

Supplementary Figure 40. GC-MS analysis after photocatalytic stability test. GC-MS spectra of the possible residual products absorbed on Cu-TiO_2 after the 24 h photoreduction $^{13}CO_2$ (Cu-TiO_2 heated for 1 h at 100 °C to desorb the adsorbate).

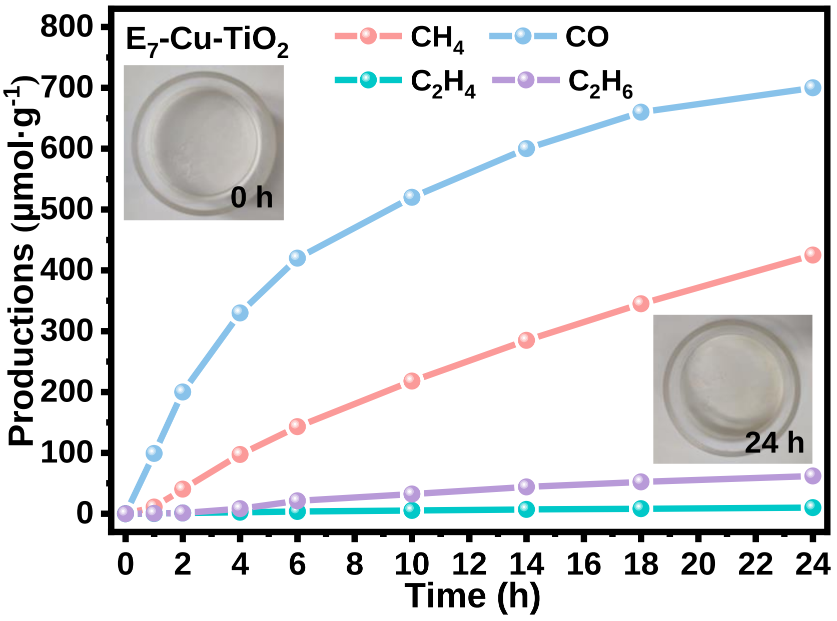

Supplementary Figure 41. Photocatalytic stability test of E_7-Cu-TiO_2. Long-time photocatalytic stability test of E_7-Cu-TiO_2.

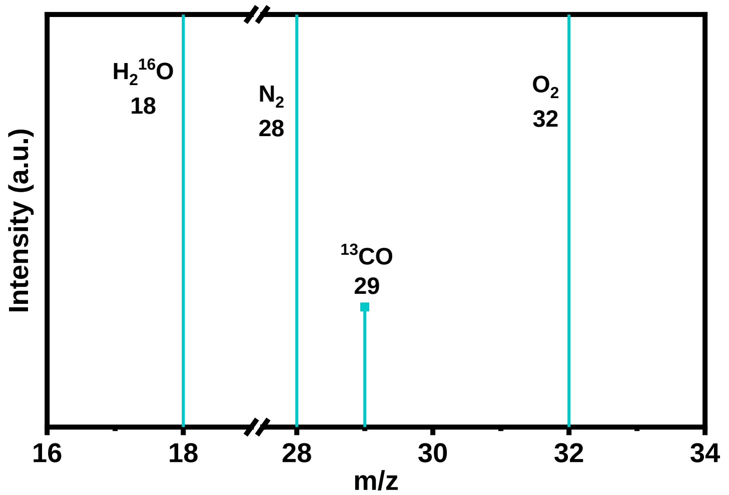

Supplementary Figure 42. GC-MS analysis after photocatalytic stability test. GC-MS spectra of the possible residual products absorbed on E_7-Cu-TiO_2 after the 24 h photoreduction $^{13}CO_2$ (E_7-Cu-TiO_2 heated for 1 h at 100 °C to desorb the adsorbate).

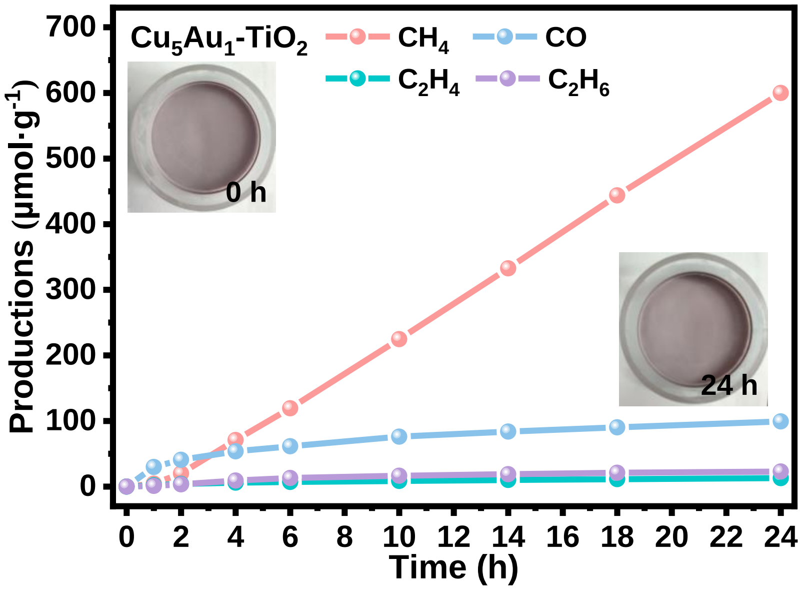

Supplementary Figure 43. Photocatalytic stability test of Cu_5Au_1-TiO_2.

Long-time photocatalytic stability test of Cu_5Au_1-TiO_2.

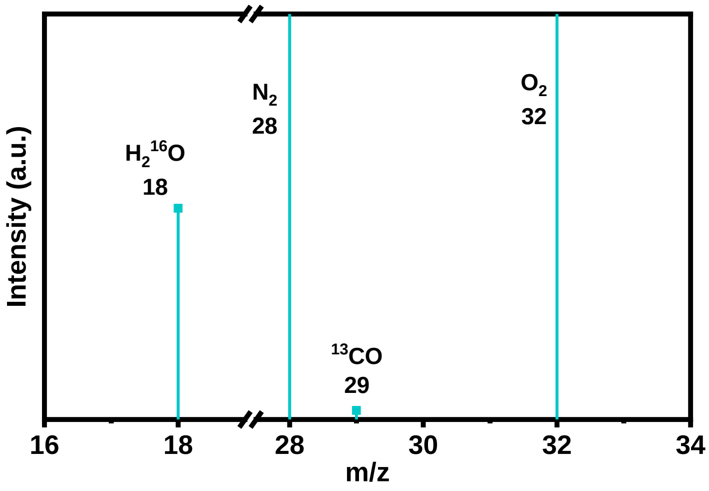

Supplementary Figure 44. GC-MS analysis after photocatalytic stability test. GC-MS spectra of the possible residual products absorbed on Cu_5Au_1-TiO_2 after the 24 h photoreduction $^{13}CO_2$ (Cu_5Au_1-TiO_2 heated for 1 h at 100 °C to desorb the adsorbate).

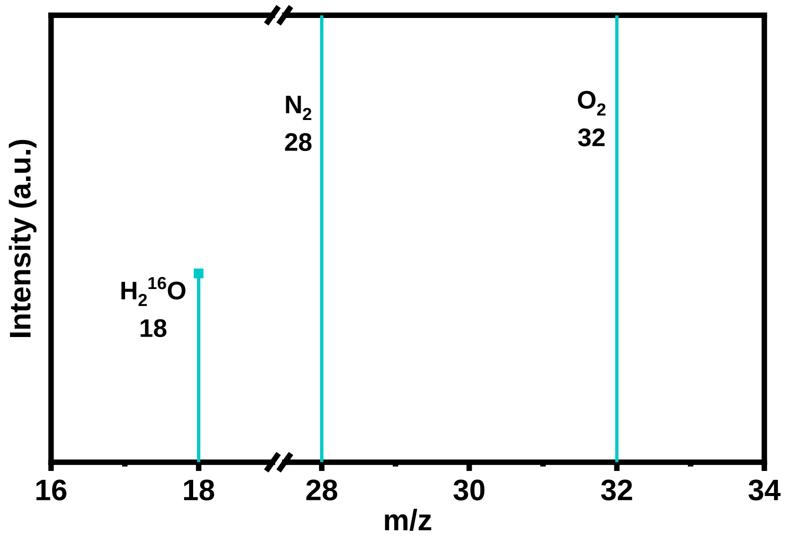

Supplementary Figure 45. GC-MS analysis after photocatalytic stability test. GC-MS spectra of the possible residual products absorbed on E_7-Cu_5Au_1-TiO_2 after the 24 h photoreduction $^{13}CO_2$ (E_7-Cu_5Au_1-TiO_2 heated for 1 h at 100 °C to desorb the adsorbate).

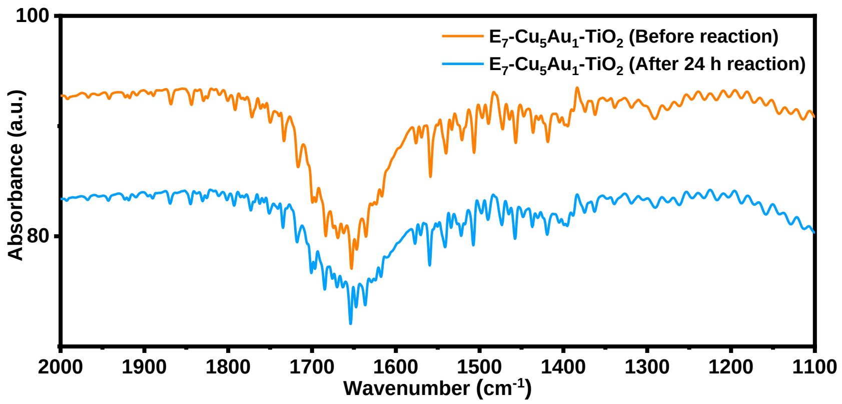

Supplementary Figure 46. FTIR analysis before and after photocatalytic stability tests. FTIR spectrum of E_7-Cu_5Au_1-TiO_2 before and after CO_2 photoreduction.

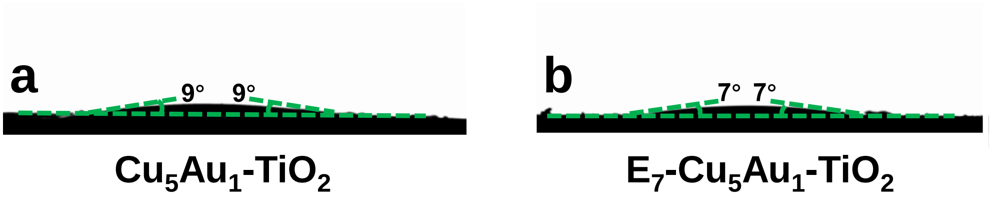

Supplementary Figure 47. Hydrophilia analysis by static water contact-angle measurement. Static water contact-angle measurement of Cu_5Au_1-TiO_2 (**a**) and E_7-Cu_5Au_1-TiO_2 (**b**).

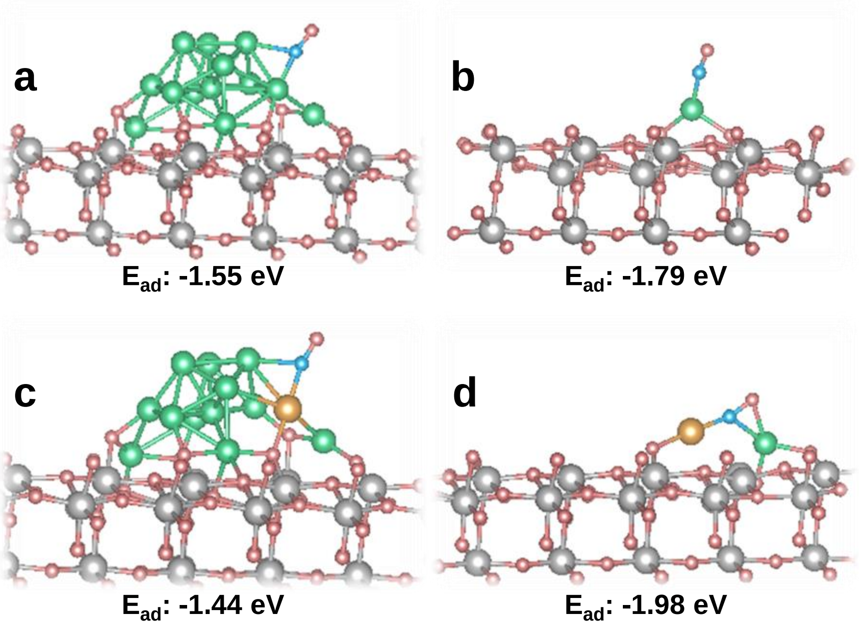

Supplementary Figure 48. Photocatalytic mechanism for the deactivation of photocatalysts. Optimized structures of CO absorbed on (**a**) Cu-NCs-TiO_2, (**b**) Cu-SAs-TiO_2, (**c**) CuAu-alloy-TiO_2, (**d**) CuAu-DAs-TiO_2, and corresponding adsorption energies.

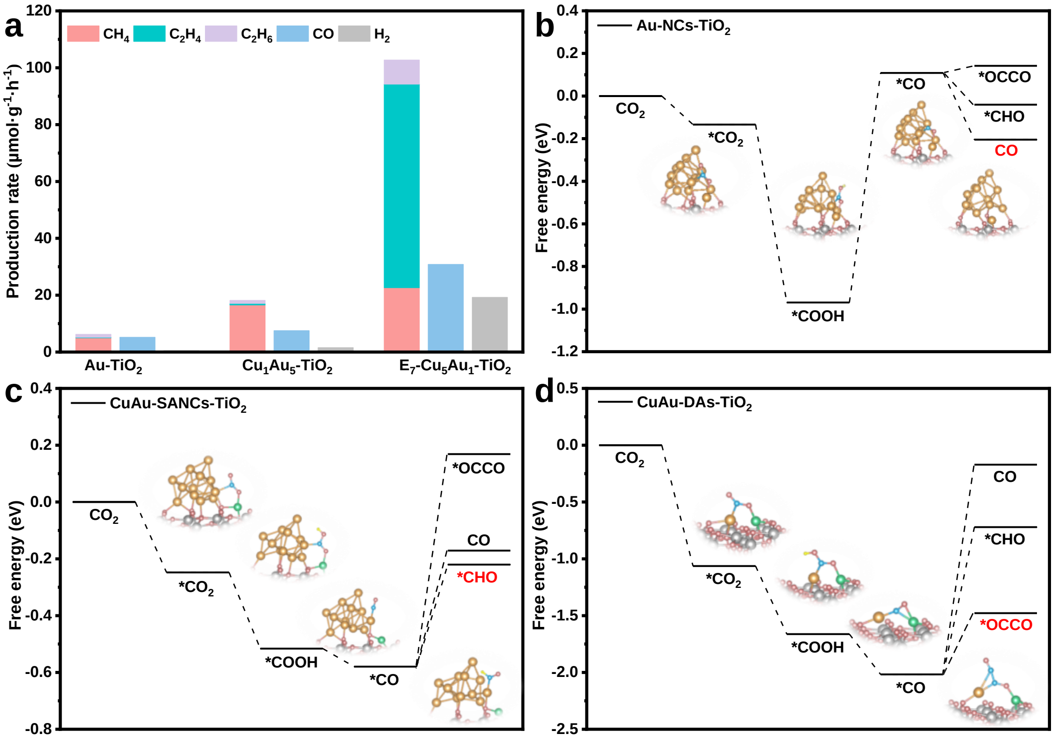

Supplementary Figure 49. Photoconversion CO_2 mechanism over different photocatalysts. **a**, CH_4, C_2H_4, C_2H_6, CO, and H_2 production rates of Au-TiO_2, Cu_1Au_5-TiO_2, and E_7-Cu_5Au_1-TiO_2. The free energy diagram of CO_2 reduction to CO, *CHO, and *OCCO over (**b**) Au-NCs-TiO_2, (**c**) CuAu-SANCs-TiO_2, and (**d**) CuAu-DAs-TiO_2 modeled surfaces.

In Supplementary Fig. 49a, only little of CO and CH_4 production are observed on pure Au nanoparticles (NPs) modified TiO_2 (Au-TiO_2) during the CO_2 reduction process, indicating that the pure Au NPs could not favor the C_2 products evolution. When few Cu species attached on Au NPs (Cu_1Au_5-TiO_2), the CH_4 production increases hugely compared to pure Au NPs, while almost no C_2 products are detected, implying that the synergistic effects between adjacent Cu atoms and Au NPs merely can promote the protonation of C_1 intermediates rather than C-C coupling

reaction. Moreover, the E_7-Cu_5Au_1-TiO_2 displays the dramatically increased C_2H_4 and C_2H_6 activity and selectivity, which uncovers the predominated C-C coupling reaction on such CuAu diatomics (DAs) structure. The density functional theory (DFT) simulations of different CuAu structures are replnished to distinguish the relationship between structure and performance, especially for the crucial step of *CO conversion. As shown in Supplementary Fig. 49b, during the possible routes of *CO conversion on pure Au nanoclusters (NCs) modified TiO_2, the CO desorption is the most energetically favorable, indicating the predominated C_1 products evolution on pure Au modified TiO_2. After the Cu single atoms (SAs) introduction in Au NCs (Supplementary Fig. 49c), the protonation of *CO is more energetically favorable on CuAu-SANCs-TiO_2 compared to the dominated CO desorption on Au-NCs-TiO_2, which further replenishs that the synergistic effects between Cu SAs and Au NCs could not promote the C-C coupling in thermodynamics. Notably, only the combination of Cu SAs and Au SAs (CuAu-DAs) could enormously decrease the C-C coupling energy barrier and turn the C-C coupling into the predominated route during the CO_2 reduction process (Supplementary Fig. 49d), which demonstrates that the C_2 products are mainly ascribed to the thermodynamic advantage of C-C coupling on CuAu-DAs structure.

Supplementary Table 1. Photoconversion CO_2 with H_2O into C_2H_4 related research.

Photocatalyst	Experimental condition	Light source	C_2H_4 yield (μmol·g^{-1}·h^{-1})	Stability (h)	Reference
CuAu-DAs-TiO_2	2 mg of catalyst, 90 KPa CO_2 and 0.5 mL of H_2O	300 W Xe-lamp	568.8	>120	This work
CuAu/TiO_2	2 mg of catalyst, 90 KPa CO_2 and 0.15 mL of H_2O	300 W Xe-lamp	369.8	20	*ACS Nano* 15, 14453-14464 (2021)
$In_{2.77}S_4$(P6)	3 mg of catalyst, CO_2 and 20 mL of H_2O	450 W Xe-lamp	67.7	5	*J. Am. Chem. Soc.* 145, 422-435 (2023)
$CuGaS_2$	5 mg of catalyst, 101 KPa CO_2 and 20 mL of H_2O	450 W Xe-lamp	20.6	14	*Angew. Chem. Int. Ed.* 63, e202216613 (2023)
V_S-$AgInP_2S_6$	4-5 mg of catalyst, 101 KPa CO_2 and 0.4 mL of H_2O vapor	300 W Xe-lamp	18.3	6	*Nat. Commun.* 12, 4747 (2021)
$Cu^{\delta+}$/CeO_2-TiO_2	10 mg of catalyst, 100 KPa CO_2 and 30 mL of H_2O	300 W Xe-lamp	4.5	5	*ACS Nano* 16, 2306-2318 (2022)
C@Fe_2C/TiO-2	10 mg of catalyst, 70 KPa CO_2 and 90 mL of H_2O	300 W Xe-lamp	4.3	4	*Chem. Eng. J.* 423, 130190 (2021)
Mo-COF	10 mg of catalyst, CO_2 and 5 mL of H_2O	300 W Xe-lamp	3.6	2	*Appl. Catal. B: Environ.* 291, 120146 (2021)
CuO_X@p-ZnO	5 mg of catalyst, 50 KPa CO_2 and H_2O vapor	300 W Xe-lamp	2.7	8	*J. Am. Chem. Soc.* 143, 2984-2993 (2021)
Co-doped NiS_2	10 mg of catalyst, 10 Kpa CO_2 and H_2O vapor	300 W Xe-lamp	2.5	10	*Nano Res.* 15, 1882-1891 (2022)
CuACs/PCN	5 mg of catalyst, 10 1 KPa CO_2 and 45 mL of H_2O	300 W Xe-lamp	1.6	3	*Adv. Mater.* 35, 2208132 (2022)
Sv-CdS@ZIF-8	20 mg of catalyst, 80 KPa CO_2 and 2 mL of H_2O vapor	300 W Xe-lamp	0.8	5	*Appl. Catal. B: Environ.* 285, 119834 (2021)

Supplementary Table 2. EXAFS fitting parameters at the Cu K-edge for various samples (S_0^2=0.845).

Sample	Shell	CN^a	R(Å)^b	σ^2(×10^{-3} Å^2)^c	ΔE_0(eV)^d	R factor
Cu foil	Cu-Cu	12*	2.54	8.5	4.0	0.003
Sample	Cu-O	3.3	1.93	4.8	5.2	0.01
	Cu-Au	1.2	2.91	7.0	9.8	

^{a}*CN*, coordination number; ^{b}*R*, distance between absorber and backscatter atoms; $^c\sigma^2$, Debye-Waller factor to account for both thermal and structural disorders; $^d\Delta E_0$, inner potential correction; *R* factor indicates the goodness of the fit. S_0^2 was fixed to 0.845, according to the experimental EXAFS fit of Cu foil by fixing *CN* as the known crystallographic value. Fitting range: $2.5 \leq k$ (/Å) ≤ 12.5 and $1.0 \leq R$ (Å) ≤ 3.0 (Cu foil); $2.0 \leq k$ (/Å) ≤ 12 and $1.0 \leq R$ (Å) ≤ 3.0 (E_7-Cu_5Au_1-TiO_2). A reasonable range of EXAFS fitting parameters: $0.700 < S_0^2 < 1.000$; $CN > 0$; $0.02 > \sigma^2 > 0.003$Å^2; $|\Delta E_0| < 10$ eV; *R* factor < 0.02.

Supplementary Table 3. The contents of metal atoms in Cu_5Au_1-based hybrid structures determined by ICP–AES.

Photocatalyst	Cu content (wt%)	Au content (wt%)	Cu molar content (%)	Au molar content (%)	Molar ratio of Cu:Au
Cu_5Au_1-TiO_2	2.70	1.66	1.12	0.22	5.04
$E_{0.5}$-Cu_5Au_1-TiO_2	1.86	1.69	0.77	0.23	3.42
E_1-Cu_5Au_1-TiO_2	1.37	1.59	0.57	0.21	2.67
E_3-Cu_5Au_1-TiO_2	1.12	1.59	0.47	0.21	2.18
E_5-Cu_5Au_1-TiO_2	0.73	1.63	0.30	0.22	1.38
E_7-Cu_5Au_1-TiO_2	0.59	1.68	0.24	0.22	1.08
E_9-Cu_5Au_1-TiO_2	0.44	1.66	0.18	0.22	0.82
E_{11}-Cu_5Au_1-TiO_2	0.30	1.65	0.13	0.22	0.57
E_{13}-Cu_5Au_1-TiO_2	0.17	1.64	0.07	0.22	0.32
$H_{0.5}$-Cu_5Au_1-TiO_2	2.51	1.67	1.04	0.22	4.67
H_1-Cu_5Au_1-TiO_2	2.31	1.62	0.96	0.22	4.41
H_3-Cu_5Au_1-TiO_2	2.31	1.64	0.96	0.22	4.36
H_5-Cu_5Au_1-TiO_2	2.26	1.63	0.94	0.22	4.31
H_7-Cu_5Au_1-TiO_2	2.21	1.61	0.91	0.22	4.25
H_9-Cu_5Au_1-TiO_2	2.26	1.64	0.94	0.22	4.27
H_{11}-Cu_5Au_1-TiO_2	2.18	1.59	0.90	0.21	4.25
H_{13}-Cu_5Au_1-TiO_2	2.24	1.65	0.93	0.22	4.22

Supplementary Table 4. The time-resolved fluorescence spectra of as-prepared samples.

Photocatalysts	A_1	τ_1/ns	A_2	τ_2/ns	A_3	τ_3/ns	τ/ns
TiO_2	730.23	1.23	209.76	6.24	168.95	47.39	38.05
Cu-TiO_2	1193.29	0.60	45.55	3.87	18.30	39.42	18.25
Cu_5Au_1-TiO_2	1038.55	1.05	115.17	4.44	39.85	35.69	17.94
E_7-Cu_5Au_1-TiO_2	1097.26	0.79	104.96	3.18	10.07	29.44	6.99

Analysis method of time-resolved PL spectra.

A “tri-exponential” function was used to fit the decay curves as follows[11,12]:

$$\text{Fit} = \text{A} + A_1 exp\left(-\frac{t}{\tau_1}\right) + A_2 exp\left(-\frac{t}{\tau_2}\right) + A_3 exp\left(-\frac{t}{\tau_3}\right)$$

The average charge carrier lifetime (τ) can be calculated from the equation as follows: [$\tau = (A_1\tau_1^2 + A_2\tau_2^2 + A_3\tau_3^2) / (A_1\tau_1 + A_2\tau_2 + A_3\tau_3)$]

Supplementary Table 5. The contents of metal atoms in $Cu_7^N Au_1$-based hybrid structures determined by ICP–AES.

Photocatalyst	Cu content (wt%)	Au content (wt%)	Cu molar content (%)	Au molar content (%)	Molar ratio of Cu:Au
$E_{0.5}$-$Cu_7^N Au_1$-TiO_2	3.54	1.55	1.47	0.21	6.73
E_1-$Cu_7^N Au_1$-TiO_2	2.32	1.59	0.96	0.21	4.53
E_3-$Cu_7^N Au_1$-TiO_2	1.93	1.64	0.80	0.22	3.66
E_5-$Cu_7^N Au_1$-TiO_2	1.39	1.62	0.58	0.22	2.66
E_7-$Cu_7^N Au_1$-TiO_2	0.85	1.68	0.35	0.22	1.57
E_9-$Cu_7^N Au_1$-TiO_2	0.59	1.62	0.25	0.22	1.14
E_{11}-$Cu_7^N Au_1$-TiO_2	0.46	1.67	0.19	0.22	0.85
E_{13}-$Cu_7^N Au_1$-TiO_2	0.34	1.61	0.14	0.22	0.66

The calculation details of product electron-based selectivity and AQY in photocatalytic CO_2 reduction on E_7-Cu_5Au_1-TiO_2.

The electron-based selectivity of E_7-Cu_5Au_1-TiO_2 was calculated according to the following equation:

$$CH_4 = \frac{8\times Y_{CH_4}}{8\times Y_{CH_4}+2\times Y_{CO}+12\times Y_{C_2H_4}+14\times Y_{C_2H_6}+2\times Y_{H_2}} \times 100\% = 14.37\% \quad (1)$$

$$CO = \frac{2\times Y_{CO}}{8\times Y_{CH_4}+2\times Y_{CO}+12\times Y_{C_2H_4}+14\times Y_{C_2H_6}+2\times Y_{H_2}} \times 100\% = 4.90\% \quad (2)$$

$$C_2H_4 = \frac{12\times Y_{C_2H_4}}{8\times Y_{CH_4}+2\times Y_{CO}+12\times Y_{C_2H_4}+14\times Y_{C_2H_6}+2\times Y_{H_2}} \times 100\% = 68.25\% \quad (3)$$

$$C_2H_6 = \frac{14\times Y_{C_2H_6}}{8\times Y_{CH_4}+2\times Y_{CO}+12\times Y_{C_2H_4}+14\times Y_{C_2H_6}+2\times Y_{H_2}} \times 100\% = 9.42\% \quad (4)$$

$$H_2 = \frac{2\times Y_{H_2}}{8\times Y_{CH_4}+2\times Y_{CO}+12\times Y_{C_2H_4}+14\times Y_{C_2H_6}+2\times Y_{H_2}} \times 100\% = 3.06\% \quad (5)$$

Where Y_{CH_4}, Y_{CO}, $Y_{C_2H_4}$, $Y_{C_2H_6}$, and Y_{CH_4} represent the production rate of CH_4, CO, C_2H_4, C_2H_6, and H_2, respectively.

AQY calculation for 8 h:

Apparent quantum yield (AQY) is defined as the ratio of number of reacted electrons to the number of incident photons. It is given by the below equation[13]:

$$AQY = \frac{\text{Number of reacted electrons}}{\text{Number of incident photons}} \times 100\%$$

$$AQY(CH_4) = \frac{8\times N_A\times N_{CH_4}}{\frac{I\times A\times t\times \lambda}{h\times c}} \times 100\%$$

Where N_A, N_{CH_4}, I, A, t, λ, h, and c are corresponded to Avogadro's number (6.022×10^{23} mol^{-1}), mole of CH_4 production, light intensity (2500 W·m^{-2}), irradiation area (12.56×10^{-4} m^2), irradiation time (8×3600 s), wavelength (350×10^{-9} m), Planck's constant (6.626×10^{-34} J·s), and speed of light (3×10^8 m·s^{-1}), respectively. Since the bandgap of TiO_2 as estimated from UV-visible DRS analysis is 3.26 eV, unbound and free electron-hole pairs cannot be generated for 380 nm. Therefore, photons with wavelength 320–380 nm can excite electrons, whose average photonic wavelength (λ = 350 nm)[14-16]. Meanwhile, the AQY calculation of CO, C_2H_4, and C_2H_6 is also similar with that of CH_4 except the different reacted electrons. For example, the AQY of E_7-C_5A_1-TiO_2 for CH_4, CO, C_2H_4, and C_2H_6 can be calculated as follow in solid-gas mode:

$$\mathrm{AQY(CH_4)} = \frac{8\times6.02\times10^{23}\times181.0\times10^{-6}}{\dfrac{2500\times12.56\times10^{-4}\times8\times3600\times350\times10^{-9}}{6.626\times10^{-34}\times3\times10^{8}}}\times 100\%$$

$$= 0.55\%$$

$$\mathrm{AQY(CO)} = \frac{2\times6.02\times10^{23}\times246.6\times10^{-6}}{\dfrac{2500\times12.56\times10^{-4}\times8\times3600\times350\times10^{-9}}{6.626\times10^{-34}\times3\times10^{8}}}\times 100\%$$

$$= 0.19\%$$

$$\mathrm{AQY(C_2H_4)} = \frac{12\times6.02\times10^{23}\times573.0\times10^{-6}}{\dfrac{2500\times12.56\times10^{-4}\times8\times3600\times350\times10^{-9}}{6.626\times10^{-34}\times3\times10^{8}}}\times 100\%$$

$$= 2.60\%$$

$$AQY(C_2H_6) = \frac{14\times6.02\times10^{23}\times67.8\times10^{-6}}{\dfrac{2500\times12.56\times10^{-4}\times8\times3600\times350\times10^{-9}}{6.626\times10^{-34}\times3\times10^{8}}}\times100\%$$

$$= 0.36\%$$

Total $AQY = AQY(CH_4) + AQY(CO) + AQY(C_2H_4) + AQY(C_2H_6) = 3.70\%$

(Over 8 h in a static system)

Supplementary References

1. Shi, W. N. et al. Controllable synthesis of Cu_2O decorated WO_3 nanosheets with dominant (0 0 1) facets for photocatalytic CO_2 reduction under visible-light irradiation. *Appl. Catal., B* **243**, 236 (2019).

2. Jo, W. K., Kumar, S., Eslava, S. & Tonda, S. Construction of Bi_2WO_6/RGO/g-C_3N_4 2D/2D/2D hybrid Z-scheme heterojunctions with large interfacial contact area for efficient charge separation and high-performance photoreduction of CO_2 and H_2O into solar fuels. *Appl. Catal., B* **239**, 586 (2018).

3. Huang, H. W. et al. Anionic group self-doping as a promising strategy: band-gap engineering and multi-functional applications of high-performance CO_3^{2-}-doped $Bi_2O_2CO_3$. *ACS Catal.* **5**, 4094 (2015).

4. Xiao, J. D. et al. Super synergy between photocatalysis and ozonation using bulk g-C_3N_4 as catalyst: A potential sunlight/O_3/g-C_3N_4 method for efficient water decontamination. *Appl. Catal., B* **181**, 420 (2016).

5. Meng, A. Y., Zhang, L. Y., Cheng, B. & Yu, J. G. Dual cocatalysts in TiO_2 photocatalysis. *Adv. Mater.* **31**, 1807660 (2019).

6. Cao, S. W. et al. 2D/2D heterojunction of ultrathin MXene/Bi_2WO_6 nanosheets for improved photocatalytic CO_2 reduction. *Adv. Funct. Mater.* **28**, 1800136 (2018).

7. Cui, X. et al. Low-temperature Ohmic contact to monolayer MoS_2 by van der Waals bonded Co/h-BN electrodes. *Nano Lett.* **17**, 4781 (2017).

8. Wan, Y. M. et al. Conductive and stable magnesium oxide electron-selective contacts for efficient silicon solar cells. *Adv. Energy Mater.* **7**, 1601863 (2017).

9. Xie, Z. K. et al. Near-infrared light-driven photocatalytic reforming lignocellulose into H_2 and chemicals over heterogeneous carbon nitride. *ACS Catal.* **13**, 13768−13776 (2023).

10. Chen, Z. H. et al. Efficient light-free activation of peroxymonosulfate by carbon ring conjugated carbon nitride for elimination of organic pollutants. *Chem. Eng. J.* **420**, 129671 (2021).

11. Samanta, S., Satpati, B. & Srivastava, R. Unrevealing the impact of Pd nanoparticles@$BiVO_4$/S-CN heterostructure on the photo-physical & opto-electronic properties for enhanced catalytic activity in water splitting and one-pot, three-step tandem reaction. *Nanoscale Adv.* **1**, 1395-1412 (2019).

12. Samanta, S., Yadav, R., Kumar, A., Sinha, A. & Srivastava, R. Surface modified C, O co-doped polymeric g-C_3N_4 as an efficient photocatalyst for visible light assisted CO_2 reduction and H_2O_2 production. *Appl. Catal. B: Environ.* **259**, 118054 (2019).

13. Si, S. H. et al. Low-coordination single Au atoms on ultrathin $ZnIn_2S_4$ nanosheets for selective photocatalytic CO_2 reduction towards CH_4. *Angew. Chem. Int. Ed.* **61**, e202209446 (2022).

14. Wang, W. N. et al. Size and Structure Matter: Enhanced CO_2

photoreduction efficiency by size-resolved ultrafine Pt nanoparticles on TiO_2 single crystals. *J. Am. Chem. Soc.* **134**, 11276–11281 (2012).

15. Ong, W. J. et al. Self-assembly of nitrogen-doped TiO_2 with exposed {001} facets on a graphene scaffold as photo-active hybrid nanostructures for reduction of carbon dioxide to methane. *Nano Res.* **7**, 1528–1547 (2014).

16. Sorcar, S. et al. CO_2, water, and sunlight to hydrocarbon fuels: a sustained sunlight to fuel (Joule-to-Joule) photoconversion efficiency of 1%†. *Energy Environ. Sci.* **12**, 2685–2696 (2019).
